# Poly(benzodifurandione) Coated Silk Yarn for Thermoelectric Textiles

**DOI:** 10.1002/advs.202406770

**Published:** 2024-08-05

**Authors:** Mariavittoria Craighero, Qifan Li, Zijin Zeng, Chunghyeon Choi, Youngseok Kim, Hyungsub Yoon, Tiefeng Liu, Przemyslaw Sowinski, Shuichi Haraguchi, Byungil Hwang, Besira Mihiretie, Simone Fabiano, Christian Müller

**Affiliations:** ^1^ Department of Chemistry and Chemical Engineering Chalmers University of Technology Göteborg 412 96 Sweden; ^2^ Laboratory of Organic Electronics Department of Science and Technology Linköping University Norrköping 60174 Sweden; ^3^ Hot Disk AB Sven Hultins gatan 9A Göteborg 41258 Sweden; ^4^ Department of Intelligent Semiconductor Engineering Chung‐Ang University Seoul 06974 Republic of Korea; ^5^ School of Integrative Engineering Chung‐Ang University Seoul 06974 Republic of Korea

**Keywords:** organic thermoelectrics, PBFDO coated silk yarn, poly(benzodifurandione), Seebeck coefficient, thermoelectric textile

## Abstract

Thermoelectric textile devices represent an intriguing avenue for powering wearable electronics. The lack of air‐stable *n*‐type polymers has, until now, prevented the development of *n*‐type multifilament yarns, which are needed for textile manufacturing. Here, the thermomechanical properties of the recently reported *n*‐type polymer poly(benzodifurandione) (PBFDO) are explored and its suitability as a yarn coating material is assessed. The outstanding robustness of the polymer facilitates the coating of silk yarn that, as a result, displays an effective bulk conductivity of 13 S cm^−1^, with a projected half‐life of 3.2 ± 0.7 years at ambient conditions. Moreover, the *n*‐type PBFDO coated silk yarn with a Young's modulus of *E* = 0.6 GPa and a strain at break of ε_break_ = 14% can be machine washed, with only a threefold decrease in conductivity after seven washing cycles. PBFDO and poly(3,4‐ethylenedioxythiophene):poly(styrenesulfonate) (PEDOT:PSS) coated silk yarns are used to fabricate two out‐of‐plane thermoelectric textile devices: a thermoelectric button and a larger thermopile with 16 legs. Excellent air stability is paired with an open‐circuit voltage of 17 mV and a maximum output power of 0.67 µW for a temperature difference of 70 K. Evidently, PBFDO coated multifilament silk yarn is a promising component for the realization of air stable thermoelectric textile devices.

## Graphical abstract

Silk is coated with the *n*‐type polymer poly(benzodifurandione) (PBFDO) resulting in conducting yarn with an extrapolated half‐life of 3.2 ± 0.7 years at ambient conditions, which enables the fabrication of air stable thermoelectric textile generators by embroidery.

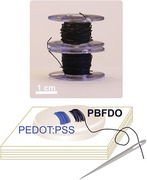

## 1 Introduction

Miniaturized and integrated electronic devices are poised to become ubiquitous in our daily lives and are increasingly used in several areas, including the Internet of Things (IoT), augmented reality (AR), robotics, health care, and wearable technology. Electronic textiles (e‐textiles) constitute a versatile platform that uses textile manufacturing technology to add new functionalities to fabrics and garments, such as health monitoring and diagnostics via sensors,^[^ ^1^, ^2^, ^3^, ^4^, ^5^ ^]^ communication via keyboards,^[^ ^6^ ^]^ displays^[^ ^7^, ^8^ ^]^ or antennae,^[^ ^9^, ^10^ ^]^ motion via actuators^[^ ^11^ ^]^ and thermal regulation via heating and cooling elements.^[^ ^12^, ^13^ ^]^ E‐textiles should also incorporate energy harvesting devices that allow the conversion of on‐site energy into electricity for powering the integrated electronics. This can include harvesting biomechanical energy through piezoelectric^[^ ^14^, ^15^ ^]^ or triboelectric generators,^[^ ^16^, ^17^ ^]^ as well as exploiting energy sources available in our surroundings, such as light through solar cells^[^ ^18^ ^]^ and heat gradients through thermoelectric devices.^[^ ^19^, ^20^, ^21^, ^22^ ^]^

Thermoelectric devices are attractive because they can harvest body heat and do not require light exposure (cf. solar cells) or motion (cf. piezo‐ and triboelectric generators). Any difference in temperature, i.e., between skin and a colder (or warmer) environment, can be directly converted into an electrical potential through the Seebeck effect. A typical thermoelectric generator (TEG) includes multiple thermocouples, each composed of two legs that are connected thermally in parallel and electrically in series. The legs of a thermocouple should be made of semiconductor materials that feature excellent thermoelectric properties, i.e., a high Seebeck coefficient α, a high electrical conductivity σ and a low thermal conductivity κ, which can be combined into a dimensionless figure of merit *ZT*:

(1) $$ZT=\frac{\alpha^{2}\sigma }{\kappa }\;T$$

where *T* is the absolute temperature. Specifically, one type of leg is composed of a *p*‐type material characterized by a positive Seebeck coefficient α_*p*_, and the other leg is composed of an *n*‐type material characterized by a negative Seebeck coefficient α_*n*_. Each pair contributes to the total open‐circuit voltage *V_oc_* of a thermoelectric generator according to:

(2) $$V_{oc}=N\left(\alpha_{p}-\alpha_{n}\right)\Delta T_{tc}$$

where *N* is the number of thermocouples and Δ*T*_*tc*_ is the temperature difference experienced by the device.^[^ ^23^ ^]^

Integrating thermoelectric devices into textiles can be achieved through multiple approaches such as 1) coating or printing a thermoelectric generator architecture onto the surface of existing fabrics,^[^ ^24^, ^25^, ^26^ ^]^ 2) stitching or embroidering conducting fibers into fabrics,^[^ ^19^, ^25^, ^27^ ^]^ and 3) knitting or weaving of the complete fabric using conducting yarns. In all cases, it is crucial to use thermoelectric materials that possess properties, which match the requirements for comfort and safety of fabrics, such as a low weight and flexibility, and the materials must not be toxic. While inorganic semiconductors offer very promising thermoelectric properties, they tend to comprise scarce and harmful elements and require energy‐intensive processing methods. Similarly, carbon nanomaterials such as carbon nanotubes and graphene are associated with toxicity concerns.^[^ ^28^ ^]^

Organic semiconductors and in particular conjugated polymers can exhibit outstanding mechanical properties and offer advantages such as ease of processing and a low thermal conductivity.^[^ ^23^, ^29^ ^]^ For instance, many conjugated polymers feature a Young's modulus of *E* = 0.01 to 1 GPa and strain at break ε_break_ > 10% (note that textile manufacturing typically requires reversible stretching to at least 5%).^[^ ^30^ ^]^ Furthermore, it is desirable that the polymer does not comprise reactive compounds such as redox dopants or strong acids, which are often used to optimize the electrical conductivity.

Extensive research has been dedicated to *p*‐type conjugated polymers, resulting in materials with promising electrical properties. One notable example is poly(3,4‐ethylenedioxythiophene):poly(styrenesulfonate) (PEDOT:PSS), which can exhibit a high σ > 1000 S cm^−1^ and overall a promising *ZT* > 0.3.^[^ ^31^, ^32^ ^]^ PEDOT:PSS can be used to wet‐spin monofilaments.^[^ ^27^, ^33^ ^]^ Despite displaying outstanding thermoelectric properties as well as a high Young's modulus, single monofilaments face some limitations due to their small cross‐sectional area, which results in a breaking force that is too low for many textile manufacturing techniques. Instead, multifilament yarns are typically used in the case of classical textile materials. Nevertheless, the preparation of multifilament PEDOT:PSS yarns has not been successful yet. Another possible approach to obtain electrically conducting multifilament yarns is coating textile yarns with conductive inks. For example, silk or cellulose yarns have been coated with PEDOT:PSS^[^ ^34^, ^35^ ^]^ and used as building blocks for the design of thermoelectric textiles through embroidery or machine sewing.^[^ ^19^, ^36^ ^]^ Nonetheless, the utility of these thermoelectric devices was limited because silver‐plated yarn had to be used to connect *p*‐type legs instead of an *n*‐type yarn due to the lack of air‐stable *n*‐type organic conductors (replacing α_*n*_ in Equation (2) with α_*Ag*_ = +0.3 µV K^−1^ limits *V_oc_*).^[^ ^19^, ^36^ ^]^ Alternatively, some studies that explore the design of thermoelectric textiles use *n*‐type yarn or monofilaments based on inorganic materials, e.g., Bi_2_Te_3_ or Ti_3_C_2_T_x_ MXene nanoflakes,^[^ ^37^, ^38^ ^]^ or carbon allotropes, such as nanotubes^[^ ^21^, ^39^ ^]^ and graphene.^[^ ^40^ ^]^ While these types of materials yield a promising thermoelectric performance, e.g., a power factor α^2^σ above 2000 µW m^−1^ K^−2^ in case of yarns based on carbon nanotubes,^[^ ^41^, ^42^ ^]^ the high mechanical stiffness and limited ductility as well as toxicity concerns may limit their use for wearable devices.

Recently, a first example of a fully polymer‐based in‐plane thermoelectric textile device was reported that utilized two sets of cellulose yarn coated with *p*‐ and *n*‐type polymer‐based materials, i.e., PEDOT:PSS and poly(benzimidazobenzophenanthroline):poly(ethyleneimine) (BBL:PEI).^[^ ^43^ ^]^ BBL:PEI is a fairly air‐stable (up to two days) *n*‐type material that is suitable for the manufacture of TEGs,^[^ ^44^ ^]^ has a σ of up to 8 S cm^−1[^ ^45^ ^]^ and a high elastic modulus, with neat BBL featuring an *E* ≈ 8 GPa.^[^ ^46^ ^]^ The BBL:PEI yarn showed an adequate mechanical robustness along with some degree of ambient stability for several days, which was achieved through encapsulation and annealing in a glovebox.^[^ ^43^ ^]^

**Figure 1:**
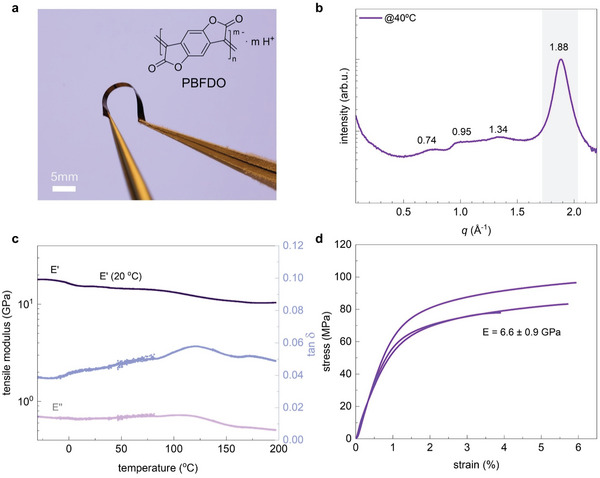
Thermomechanical properties of PBFDO films. a) Chemical structure of reduced PBFDO and photograph of a bent film after being immersed in liquid nitrogen for a few minutes; b) transmission WAXS diffractograms of a free‐standing film cast at 40 °C; c) DMTA thermographs of a free‐standing film showing the storage modulus *E*′, loss modulus *E*′′ and loss tangent tan δ (see Figure S4, Supporting Information for additional measurements); and d) stress–strain response of three free‐standing films measured by tensile deformation at 20 °C (processed and measured in the same way, i.e., drop casting at 40 °C, force rate of 0.01 N min^−1^).

To enable the design of truly functional polymer‐based thermoelectric textile devices, it will be necessary to develop an *n*‐type yarn with greatly improved air stability, mechanical robustness as well as processability. Recently, a new *n*‐type polymer, poly(benzodifurandione) (PBFDO) (see **Figure** **1**a for chemical structure), with a high electrical conductivity of σ > 1000 S cm^−1^ has been reported.^[^ ^47^, ^48^ ^]^ Wet‐spun monofilaments of PBFDO feature a similar value of σ ≈ 1000–1600 S cm^−1^, which dropped by a factor of two after 14 days, and a high *E*  =  19.5 GPa while maintaining an ε_break_ of ≈8%.^[^ ^49^ ^]^ A remaining question is the mechanical response of PBFDO at different temperatures since the embrittlement at low temperatures would complicate its use as a textile material. Moreover, a multifilament PBFDO yarn or a coated yarn would be needed to facilitate the use of textile manufacturing techniques.

Here, we investigate the mechanical properties of PBFDO to assess its suitability as a yarn coating material. We find that PBFDO can be easily processed into bulk films that remain bendable and feature some degree of stretchability even at temperatures below room temperature. Its processability and outstanding mechanical properties allow us to prepare an *n*‐type conducting yarn by coating silk yarn with PBFDO. The resulting *n*‐type multifilament yarn features a similar bulk electrical conductivity as PEDOT:PSS coated yarn and, crucially, a very promising stability for at least 14 months at ambient conditions without encapsulation. Moreover, the PBFDO coated yarn can be machine washed at least seven times. Further, the PBFDO coated yarn can be readily used to sew a button and construct a textile thermoelectric device through embroidery, which demonstrates that the yarn exhibits the robustness that is required for textile manufacturing.

## 2 Results and Discussion

In a first set of experiments, we measured the electrical conductivity of spin‐coated films with a thickness of 10 nm. The conductivity increases with temperature starting from a value of σ = 1000 S cm^−1^ at −200 °C to 1600 S cm^−1^ at 130 °C (Figure S1, Supporting Information). The observed electrical conductivity is similar to previously reported values for thin films (2000 S cm^−1^)^[^ ^47^ ^]^ and wet‐spun monofilaments (1000 to 1600 S cm^−1^ depending on the draw ratio),^[^ ^49^ ^]^ which indicates that the here studied PBFDO batch is of comparable quality.

We then investigated the thermomechanical properties of free‐standing films of PBFDO. PBFDO in dimethyl sulfoxide (DMSO) was drop‐cast at 40 °C onto glass slides to prepare films with a thickness ranging from 7 to 18 µm, which could be peeled off to obtain free‐standing films (see Experimental Section for details). The resulting free‐standing PBFDO films were mechanically robust, and could be handled without fracture, even when cooled to below room temperature. Bending without breaking was possible after being immersed in liquid nitrogen for a few minutes (bending radius 5 mm; Figure 1a; Figure S2, Supporting Information). However, thermal annealing, e.g., at 200 °C for 5 min (chosen because the polymer is stable up to 250 °C according to thermogravimetric analysis, TGA; see Figure S3, Supporting Information), resulted in brittle samples that fractured upon bending, which complicated their handling. Evidently, excessive thermal annealing should be avoided to preserve the mechanical robustness of PBFDO. The free‐standing PBFDO films featured an electrical conductivity of σ = 627 S cm^−1^ at room temperature, which is lower than the value measured for spin‐coated films (Figure S1, Supporting Information). We tentatively assign the difference in electrical conductivity to subtle variations in nanostructure as a result of different processing techniques, i.e., spin coating versus drop casting.

We carried out transmission wide‐angle X‐ray scattering (WAXS) to investigate the nanostructure of free‐standing PBFDO films. The WAXS diffractograms feature a distinct peak at *q*_010_ = 1.88 Å^−1^ (Figure 1b), which we assign to *π*–*π* stacking of the polymer backbone in accordance with a previous report.^[^ ^47^ ^]^

Dynamic mechanical thermal analysis (DMTA) in tensile mode at a frequency of 1 Hz was used to investigate the thermomechanical behavior of free‐standing PBFDO films. The tensile storage modulus *E*′ has a value of 18 GPa at −10 °C and slightly decreases with temperature, reaching a value of *E*′ = 16 GPa at room temperature and 10 GPa at 200 °C (Figure 1c and **Table** **1**; Figure S4, Supporting Information). Evidently, PBFDO maintains its stiffness up to the onset of degradation at T > 200 °C, as inferred from TGA (Figure S3, Supporting Information).

**Table 1:** Elastic modulus and strain at break ε_break_ of free‐standing PBFDO films at −10 and 20 °C (room temperature) measured by tensile drawing in force‐controlled mode using a rate of 0.01 N min^−1^ and DMTA in tensile mode at 1 Hz (mean values and standard deviation of measurements of 2–3 samples; ^a^single measurement).

	−10 °C		20 °C	
Technique	Elastic modulus [GPa]	ε_break_ [%]	Elastic modulus [GPa]	ε_break_ [%]
Tensile drawing	^a^15	^a^2.8	6.6 ± 0.9	5.2 ± 1.1
DMTA	18 ± 0.9	–	16 ± 1	–

We used tensile deformation of free‐standing samples to analyze the stiffness and ductility of PBFDO in more detail. At room temperature, the polymer is stiff but relatively ductile, with a Young's modulus *E* = 6.6 ± 0.9 GPa and a strain at break ε_break_ = 5.2 ± 1.1% when measured in force‐controlled mode with a rate of 0.01 N min^−1^ (Figure 1d and Table 1), yielding a toughness of 3.6 ± 1.1 MJ. Cooling resulted in a stiffer but more brittle material, as indicated by tensile deformation at −10 °C, which yielded a high Young's modulus of 15 GPa but low ε_break_ of only 2.8% (Table 1; Figure S5, Supporting Information). We explain the somewhat lower Young's modulus values compared with the storage modulus from DMTA with the here employed low tensile deformation rate.

Given that many textile manufacturing processes require materials with at least some degree of mechanical robustness (e.g., bendable and reversibly stretchable to 5%),^[^ ^30^ ^]^ we decided to use PBFDO in its as‐cast state as a coating material. A batch coating process was used to prepare conducting *n*‐type yarn, which involved two cycles of immersion of silk yarn in the PBFDO:DMSO ink followed by drying at 40 °C (see Experimental Section for details). Using this method, we produced continuous conductive yarn with a length of up to 1.5 m that comprised ≈10 wt.% of PBFDO (measured by weighing yarn before and after coating).

**Figure 2:**
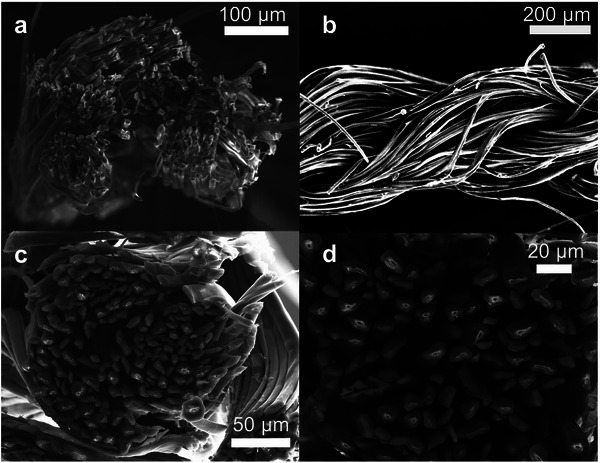
SEM micrographs of a) the cross section of neat and b) a side view of PBFDO coated silk yarn sputtered with gold, and c,d) the cross section of silk yarn coated with PBFDO (no gold sputtering).

Scanning electron microscopy (SEM) was used to assess the quality of the PBFDO coating (**Figure** **2**). SEM images of the yarn surface and cross section of freeze‐fractured yarn sections do not reveal any outer shell layer (Figure 2b–d). Intriguingly, charging artifacts occur within the inner part of each filament, which indicate an insulating silk core surrounded by a conductive outer layer. We argue that PBDFO is present on the surface of individual filaments and may have in part ingressed into each filament. The presence of PBFDO on the silk filaments was confirmed visually by the black color upon coating and by transmission WAXS diffractograms, where a shoulder at *q*_010_ = 1.88 Å^−1^, i.e., the diffraction peak assigned to the *π*–*π* stacking of the conjugated backbone, is observed in case of the coated but not the neat silk yarn (Figure S6, Supporting Information). To understand whether electrostatics govern the interaction between PBFDO and fibroin, the main component of degummed silk, we sonicated yarn sections suspended in water with a pH ranging from 2 to 7. Since fibroin has an isoelectric point at pH 4,^[^ ^34^ ^]^ we would expect that the degree of binding strongly varies as a function of pH if electrostatic interactions dominated, as previously observed for a thiophene based conjugated polyelectrolyte.^[^ ^50^ ^]^ However, we find that sonication resulted in a significant decrease in electrical conductivity by at least two orders of magnitude, independent of the pH (Figure S7, Supporting Information). Thus, we conclude that physisorption of PBFDO onto silk filaments is not driven by electrostatic interactions.

**Figure 3:**
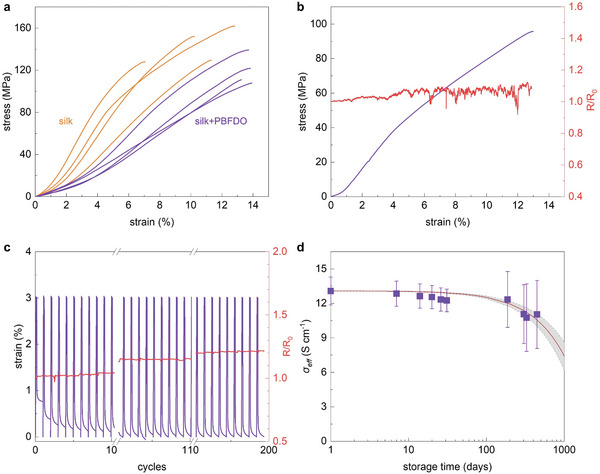
Mechanical and electrical properties of PBFDO coated silk yarn. a) Stress–strain curves recorded during tensile deformation of neat (orange) and PBFDO coated (purple) silk yarn; b) stress–strain curve of the PBFDO coated silk yarn (left) and in situ recorded change in electrical resistance *R*/*R*_0_ where *R*_0_ is the resistance of the yarn prior to the fatigue test; c) strain during cyclic tensile deformation of the PBFDO coated silk yarn repeatedly stretched to 3% then released for 60 s (purple line) together with the in situ recorded change in electrical resistance (red line); and d) effective electrical conductivity σ_eff_ of PBFDO coated yarn (purple squares) and aging prediction (grey area) as a function of storage time at ambient conditions.

We proceeded with the mechanical and electrical characterization of the conducting yarn. Tensile drawing of both neat silk yarn and yarn coated with PBFDO was carried out to explore the influence of the coating on the mechanical properties (**Figure** **3**a). The recorded stress–strain curves indicate that immersing silk yarn in PBFDO:DMSO ink reduces the Young's modulus from *E* = 1.7 ± 0.5 GPa, measured for neat silk, to 0.6 ± 0.1 GPa after coating the silk with PBFDO:DMSO ink. Simultaneously, the strain at break increased reaching a value of up to ε_break_ = 14 ± 0.3% (**Table** **2**). Similar changes are seen when immersing silk yarn in DMSO, indicating that the solvent and not the PBFDO causes the observed changes in mechanical properties (Figure S8 and Table S1, Supporting Information). We monitored the electrical resistance of the *n*‐type yarn during stretching to investigate the impact of plastic deformation beyond the yield point. We found that the normalized resistance *R*/*R*_0_, where *R*_0_ is the resistance prior to tensile deformation, remains largely unaffected until the yarn fractures (Figure 3b; Figure S9, Supporting Information).

**Table 2:** Mechanical and thermoelectric properties of coated silk yarns. Young's modulus *E*, strain at break ε_break_, effective electrical conductivity σ_eff_, Seebeck coefficient α, and power factor α^2^σ_eff_; values represent the mean and standard deviation of measurements of 5 samples; ªvalues from ref. [51].

Yarn	*E* [GPa]	ε_break_ [%]	σ_eff_ [S cm^−1^]	α [µV K^−1^]	α^2^σ_eff_ [µW m^−1^ K^−2^]
Silk	1.7 ± 0.5	10 ± 2.5	–	–	–
PBFDO coated silk	0.6 ± 0.1	14 ± 0.3	13 ± 1	−18.8 ± 0.8	0.46 ± 0.04
PEDOT:PSS coated silk	2.9 ± 0.6ª	10 ± 0.3ª	13 ± 2	17.9 ± 0.1	0.42 ± 0.06

To further evaluate how the PBFDO coated yarn responds to repeated mechanical stress, we conducted a series of fatigue tests. The resistance measured in situ during cyclic tensile deformation to 3% strain increased by less than 20% after 200 cycles (Figure 3c). PBFDO coated yarn can also withstand cyclic stretching to 4% or 5% strain without breaking for up to 60 cycles or 30 cycles, respectively, which suggests that the yarn is suitable for textile manufacturing.^[^ ^30^ ^]^ At the same time the resistance increased by only 10% or 30% (Figure S10, Supporting Information).

We investigated the electrical properties of the *n*‐type yarn by determining the effective electrical conductivity σ_eff_, which is based on the total cross‐sectional area of the yarn including the silk, PBFDO as well as voids, and corrected for the contact resistance by using the transmission line method (see Experimental Section for details). We obtained a value of σ_eff_ = 13 ± 1 S cm^−1^ for the PBFDO coated silk yarn (Table 2). Considering that PBFDO comprises 10 wt.% of the yarn and taking into account that the coated yarn comprises ≈27.5% voids (obtained by comparing the weight and volume of neat silk yarn with the density of silk ρ ≈ 1.3 g cm^−3^), the coating has an effective conductivity *σ* ≈ 250 S cm^−1^, which is lower than the value measured for PBFDO films on glass (Figure S1, Supporting Information) or free‐standing films. We attribute this difference to an uneven PBFDO coating on the filaments, resulting in an overall reduction of the electrical conductivity. We also measured the Seebeck coefficient α of the *n*‐type yarn and obtained a value of α_*n*_ = −18.8 ± 0.8 µV K^−1^ (Table 2), which is similar to the value previously reported for PBFDO thin films.^[^ ^47^ ^]^ The measured electrical conductivity and Seebeck coefficient result in a thermoelectric power factor of $\alpha_{n}^{2}\sigma_{eff}$ *=* 0.46 ± 0.04 µW m^−1^ K^−2^, which is comparable to the power factor of PEDOT:PSS coated silk yarn.^[^ ^19^ ^]^

In a further set of experiments, we assessed to which extent the thermoelectric properties of PBFDO coated silk yarn can be optimized through partial oxidation. Ke et al. have recently reported that PBFDO can be oxidized by exposing the polymer to strong oxidizing agents such as tris(4‐bromophenyl)ammoniumyl hexachloroantimonate (Magic Blue).^[^ ^52^ ^]^ We submerged PBDFO coated yarn sections in solutions of 0–3 mm Magic Blue dissolved in degassed acetonitrile and subsequently measured the electrical conductivity and Seebeck coefficient (Figure S11, Supporting Information). As anticipated, oxidized yarn sections featured a σ_eff_ that decreased with Magic Blue concentration, while α became more negative (Figure S11, Supporting Information). Overall, the power factor increased by up to one order of magnitude from ≈0.5 to 5 µW m^−1^ K^−2^ for an intermediate Magic Blue concentration of 0.25 mm. However, the thermoelectric properties of partially oxidized PBFDO yarn were not stable at ambient conditions, which we ascribe to gradual reduction of the material. Thus, we opted to use fully reduced PBFDO coated yarn for all further studies since their thermoelectric properties were stable with time.

**Figure 4:**
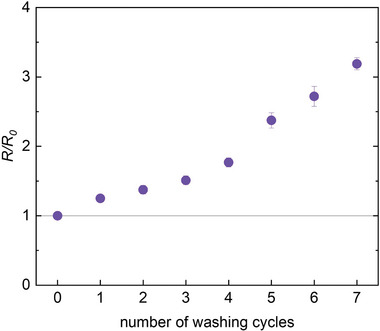
Machine‐washing of PBFDO coated silk yarn. Change in electrical resistance *R*/*R*_0_ as a function of washing cycle at 20 °C.

To assess the long‐term stability of PBFDO coated yarn, we repeatedly characterized the electrical resistance during storage at ambient conditions and, in a separate set of experiments, exposed the yarn to machine washing. Notably, the electrical conductivity remains almost constant over a span of 14 months when stored at ambient conditions (Figure 3d), which indicates exceptional stability of the yarn compared to other types of *n*‐type polymers. For instance, BBL:PEI coated regenerated cellulose yarn showed a tenfold increase in resistance after only one day at ambient conditions.^[^ ^43^ ^]^ We extrapolate that the PBFDO coated yarn will lose 50% of its initial conductivity after 3.2 ± 0.7 years (Figure S12, Supporting Information). Strikingly, the PBFDO coated yarn also featured a promising degree of washability. Seven washing cycles at 20 °C resulted in an increase in electrical resistance by a factor of only 3 (**Figure** **4**). The decrease in electrical conductivity observed upon sonication of PBFDO coated yarn (cf. Figure S7, Supporting Information) suggests that mechanical wear limits the stability during machine washing. We chose to study the PBFDO coated yarn without a protective coating to obtain a fair assessment of its stability. In the future, it would be possible to add a protective coating to the yarn such as a thermoplastic elastomer to minimize mechanical wear during washing.

**Figure 5:**
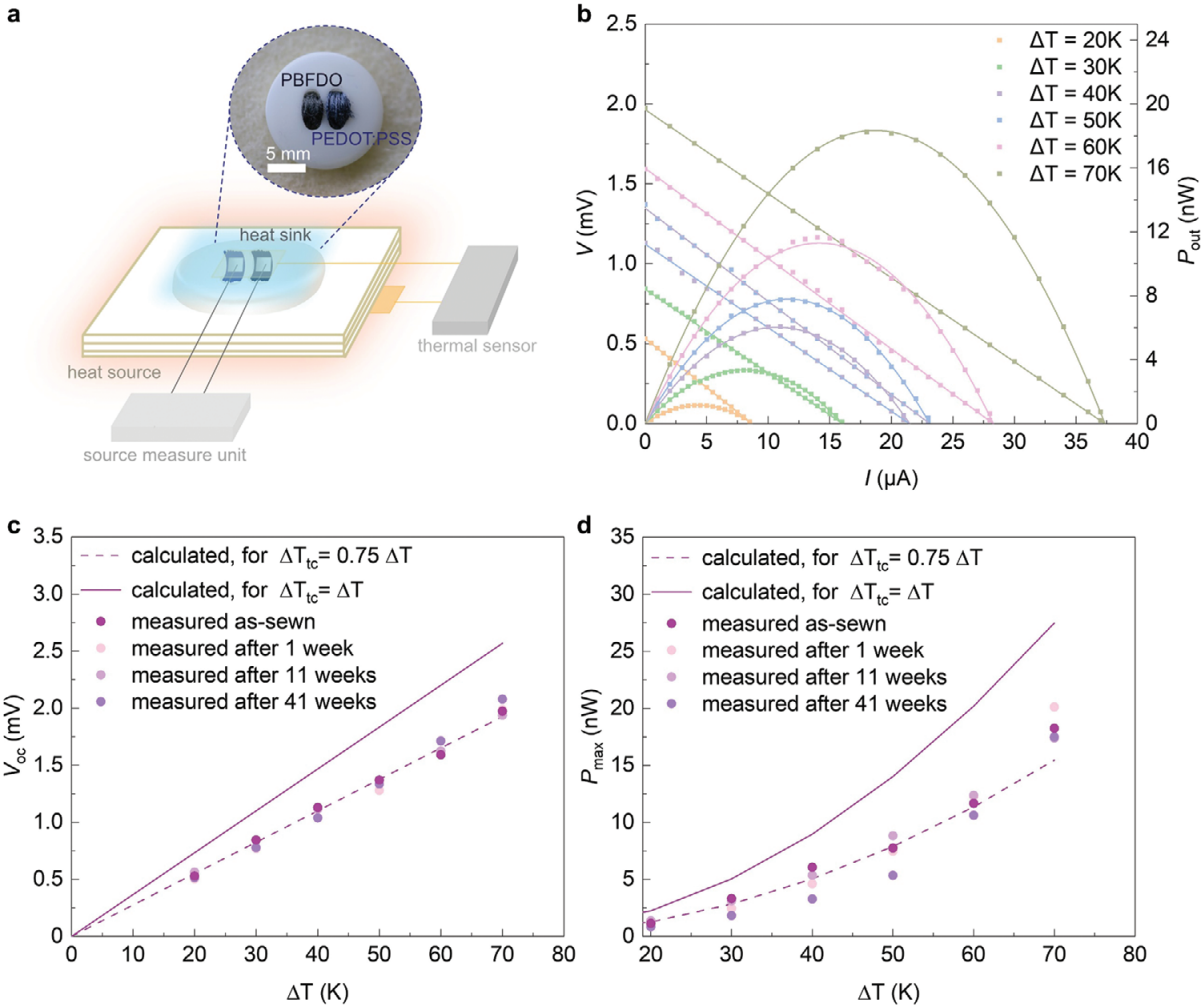
Performance of the thermoelectric button. a) Schematic representation of the thermocouple fabricated by hand‐stitching a button onto a wool fabric with the *n*‐type leg composed of PBFDO coated yarn and the *p*‐type leg of PEDOT:PSS coated yarn; b) voltage *V* (left) and output power *P*_out_ (right) of the thermoelectric button as a function of current *I* for different temperature differences Δ*T*  = *T*_hot_  − *T*_cold_ where *T*_hot_ and *T*_cold_ are the temperatures of the hot plate and cooler measured with a pair of thermocouples; c) open‐circuit voltage *V_oc_* and d) maximum output power *P*_max_ as a function of Δ*T* recorded immediately after device fabrication or after storage at ambient conditions for 1, 11 or 41 weeks (circles represent experimental values; solid and dashed lines calculated assuming that Δ*T_tc_* =  Δ*T* and Δ*T_tc_* =  0.75 · Δ*T*, respectively).

An out‐of‐plane textile thermocouple was fabricated by hand‐stitching a button with conducting yarn onto three layers of a felted wool fabric (**Figure** **5**a; Figure S13, Supporting Information). We used a button to demonstrate the ease of handling and robustness of the *n*‐type yarn. Furthermore, the button increases the length of the thermoelectric legs, thereby enhancing the thermal gradient experienced by the thermocouple. The *n*‐ and *p*‐type leg where constructed with PBFDO and PEDOT:PSS coated silk yarn, respectively, with the latter produced with a previously reported roll‐to‐roll coating method that yielded machine‐washable *p*‐type conducting yarn.^[^ ^51^ ^]^ The thermoelectric properties of the *n*‐ and *p*‐type yarns closely match, except for the opposite sign of the Seebeck coefficient (Table 2).

We characterized the performance of the thermoelectric button by placing the device between a hot plate and a heat sink with a temperature *T_hot_* and *T_cold_*, respectively (Figure 5a, see Experimental Section for details). The temperature of the hot plate was increased to generate stepwise increasing temperature gradients Δ*T*  = *T_hot_*  − *T_cold_* thus exposing the thermoelectric legs to a temperature gradient Δ*T*_*tc*_ (note that Δ*T*_*tc*_ < Δ*T* because of thermal contact resistance between the device and the heat source and sink). At each Δ*T*, the generated voltage *V* was recorded while a variable load was applied by drawing different currents *I* with a source‐measure unit.

The open‐circuit voltage *V_oc_*, i.e., the extrapolated voltage at *I* = 0, increased linearly with Δ*T* (Figure 5c) and we estimate a value of *V_oc_*/Δ*T* = 27.5 µV K^−1^ assuming that Δ*T*_*tc*_ =  Δ*T*. Instead, Equation (2) predicts a higher value of *V_oc_*/Δ*T*_*tc*_ = 36.7 µV K^−1^, which we explain with thermal contact resistance and hence Δ*T*_*tc*_ ≈ 0.75 · Δ*T* (cf. solid and dashed lines in Figure 5c). The output power was calculated according to:

(3) $$P_{\mathrm{out}}=VI$$

and reached a maximum for the current at which the internal resistance of the device *R*_in_ = 50 Ω and the load *R*_load_ are equal. The maximum output power *P*_max_ achieved by the thermocouple increased with Δ*T*, reaching a highest value of *P*_max_ = 20 nW and a power density of ≈10 nW cm^−2^ at Δ*T* = 70 K (Figure 5b,d; the projected area of the button was 1.77 cm^−2^). The measured values are in good agreement with *P*_max_ predicted according to:

(4) $$P_{\max}=\frac{V_{oc}^{2}}{4R_{in}}\;$$

which, e.g., yields a value *P*_max_ = 22 nW for *V_oc_* = 2.1 mV at Δ*T* = 70 K.

To assess the long‐term stability of the thermoelectric button, we repeatedly measured its performance. Both, *V_oc_* and *P*_max_ did not markedly change after at least 41 weeks of storage at ambient conditions, apart from the occasional device characterization during which the device was heated (Figure 5c,d; Figure S14, Supporting Information).

**Figure 6:**
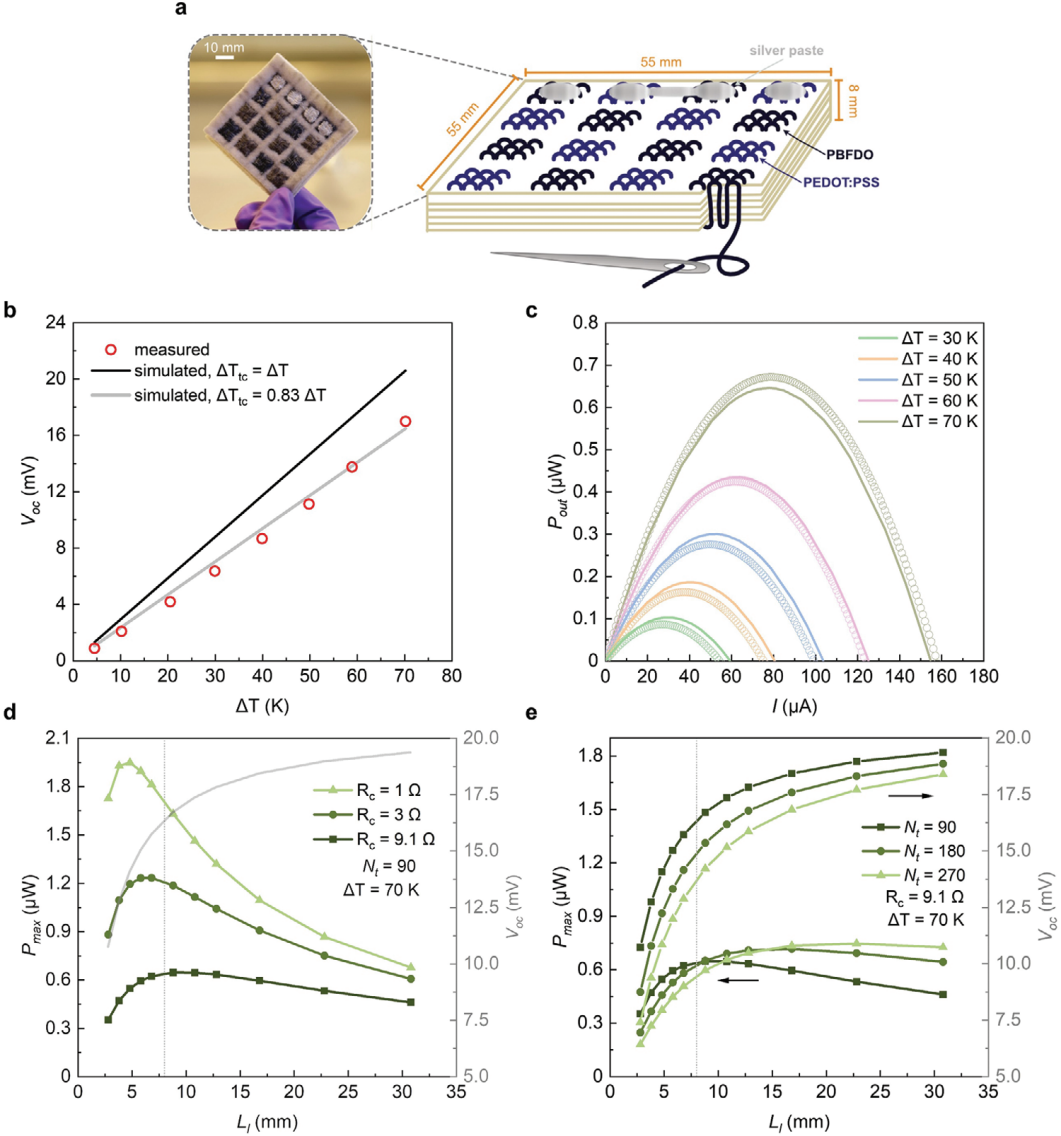
Performance of the thermoelectric generator composed of eight thermocouples. a) Schematic and photograph of the thermoelectric generator. b) Open‐circuit voltage *V_oc_* as a function of ∆*T* (circles represent experimental values; black and grey lines calculated assuming that Δ*T_tc_* =  Δ*T* and Δ*T_tc_* =  0.83 · Δ*T*, respectively); c) output power *P*_out_ of the thermopile as a function of current *I* for different temperature differences Δ*T* (circles represent experimental values, lines represent the results from the simulation at different Δ*T* considering a thermal contact resistance of 97 K cm^2^ W^−1^). d) Predicted *P*_max_ (right) and *V_oc_* (left) generated from the thermoelectric generator at Δ*T* = 70 K and thermal contact resistance of 97 K cm^2^ W^−1^ as a function of thermoelectric leg length (*L_l_*) (vertical grey line at *L_l_* = 8 mm represents the actual *L_l_* of the device). The electrical contact resistance *R_c_* per thermocouple was 1 Ω (triangles), 3 Ω (circles), 9.1 Ω (squares) (which corresponds to the actual *R_c_* of the device at Δ*T* = 70 K), and the thread count *N_t_* was set to be 90. e) Predicted *P*_max_ (right) and *V_oc_* (left) generated from the thermoelectric generator at Δ*T* = 70 K and thermal contact resistance of 97 K cm^2^ W^−1^ as a function of *L_l_* (vertical grey line at *L_l_* = 8 mm represents the actual *L_l_* of the device). The electrical contact resistance *R_c_* per thermocouple was 9.1 Ω (which corresponds to the actual *R_c_* of the device at Δ*T* = 70 K), and the thread count *N_t_* was 90 (squares), 180 (circles) and 270 (triangles).

In a further set of experiments, a larger out‐of‐plane textile thermoelectric generator was fabricated. PBFDO and PEDOT:PSS coated silk yarns were hand‐sewn through six layers of felt wool forming 8 n‐legs and 8 p‐legs, respectively (**Figure** **6**a **;** Figure S15, Supporting Information). We chose to construct *n*‐ and *p*‐type legs with the same number of yarn sections and cross‐sectional area because of the similar σ_eff_ of the two types of coated silk yarn (see Table 2), meaning that the performance of the TEG is optimized if the legs have comparable dimensions.^[^ ^19^, ^44^ ^]^

We characterized the thermoelectric performance of the generator similar to the thermoelectric button, e.g., by placing the device between a hot plate and a heat sink and by recording the generated voltage *V* while drawing different currents *I* with a source‐measure unit at each Δ*T* of interest. Moreover, a numerical simulation model was developed to investigate the impact of the thermoelectric generator design parameters on *P*_max_. In our simulation, each thermoelectric leg is depicted as a cubic structure with effective electrical and thermal conductivity (see Supporting Information for further details). We simulated the temperature distribution across the textile thermopile and the resulting *V_oc_* at fixed values of Δ*T* and took into account the thermal contact resistance. *P*_max_ was subsequently calculated using Equation (4) where the measured internal resistance *R_in_* was 119 Ω at room temperature, decreasing to 108 Ω at Δ*T* = 70 K (Figure S16, Supporting Information).

The measured open circuit voltage *V_oc_* increased linearly with Δ*T* (Figure 6b) and we estimate a value of *V_oc_*/Δ*T* = 243 µV K^−1^ assuming that Δ*T*_*tc*_ =  Δ*T*. In agreement with the measured results, the simulated *V_oc_* exhibits a linear increase with Δ*T*. By assessing the ratio between the measured *V_oc_* and the simulated *V_oc_* under ideal conditions where Δ*T_tc_* =  Δ*T*, we estimate that the thermal contact resistance constitutes 17% of the total thermal resistance of the entire setup, corresponding to a total thermal contact resistance of 97 K cm^2^ W^−1^. The measured *P*_max_ achieved by the device increased with Δ*T*, reaching a highest value of *P*_max_ = 0.67 µW at Δ*T* = 70 K. The value predicted by the simulation agrees with the experimental results (Figure 6c). The thermoelectric module with a total area of 30.25 cm^2^ yields a power density of 22 nW cm^−2^ for ∆*T* = 70 K, which is in good agreement with the power density generated by the button thermocouple considering the different device thickness and area fill factor. The thermoelectric performance of the device is unvaried after 10 days and decreased by 30% after 90 days of storage at ambient conditions (Figure S17, Supporting Information).

Furthermore, the validated model was used to investigate the impact of the length of thermoelectric legs *L_l_*, i.e., the thickness of the device, and the electrical contact resistance *R_c_* of each thermocouple on the performance of the textile device (Figure 6d). A larger *L_l_* results in a higher *V_oc_* but also leads to an increase in the internal resistance of the thermoelectric device. The interplay between *L_l_* and *R_c_* determines the optimal *L_l_* for which the highest *P*_max_ is achieved. For instance, when *R_c_* = 9.1 Ω, i.e., the actual value calculated for our device, the optimal *L_l_* is 8 mm, resulting in *P*_max_ = 0.65 µW. This aligns with the measured *P*_max_ = 0.67 µW, indicating a good agreement between the numerical model and the experimental data. Additionally, the impact of *R_c_* on the resulting output power is significant. The maximum output power reaches a value of *P*_max_ = 1.95 µW when *R_c_* = 1 Ω, exceeding the value achieved for *R_c_* = 9.1 Ω by more than three times. This substantial improvement in the highest *P*_max_ suggests that reducing *R_c_* is an effective means of enhancing the performance of the textile thermopile.

The influence of the thread count *N_t_* for each thermocouple leg was also investigated (Figure 6e). As the thermal conductivity of coated yarns is higher than that of the felted wool (see Experimental Section and ref. [19]), a higher *N_t_* means a higher portion of yarns and a higher effective thermal conductivity of the leg, which decreases the temperature gradient experienced by each leg and hence reduces *V_oc_*. On the other hand, a higher portion of yarns greatly decreases the internal electrical resistance of the textile thermopile, leading to a slightly higher *P*_max_. If *N_t_* is tripled to 270, a *P*_max_ of 0.75 µW is predicted for *R_c_* = 9.1 Ω, which is only 15% larger than the *P*_max_ for a device with a *N_t_* = 90.

In a final set of experiments, we monitored the thermoelectric performance of the textile device upon repeated bending around a coffee mug with a diameter of 10 cm. We measured the internal electrical resistance of the device before and after the mechanical deformation. The internal resistance remained largely unaffected by bending and unbending cycles, with variations of less than 2.5% compared to the initial value after 12 cycles (Figure S18, Supporting Information). Then, we tied the thermoelectric generator around the coffee mug and filled the mug with hot water to create a temperature difference across the device, i.e., between the wall of the mug and the surrounding air. When the temperature difference stabilized at Δ*T* = 30 K, we measured the thermoelectric performance of the bent device. Gratifyingly, we observed that repeated bending did not affect the thermoelectric performance of the device, which continued to function as before the mechanical deformation was applied (Figure S18, Supporting Information).

## 3 Conclusion

The *n*‐type conjugated polymer PBFDO could be readily processed into free‐standing films, exhibiting a high stiffness and relatively high ductility with a Young's modulus *E* = 6.6 ± 0.9 GPa and a strain at break ε_break_ = 5.2 ± 1.1% at room temperature. The high degree of mechanical robustness of as‐cast PBFDO enabled the fabrication of *n*‐type yarn by batch coating silk yarn with PBFDO:DMSO ink. The resulting multifilament *n*‐type yarn featured an effective electrical conductivity of σ_eff_ = 13 ± 1 S cm^−1^, which remained nearly constant over 14 months of storage at ambient conditions and increased only threefold after seven washing cycles, indicating both outstanding long‐term stability and washability.

Moreover, the PBFDO coated yarn exhibited a Young's modulus *E* = 0.6 ± 0.1 GPa and a strain at break of ε_break_ = 14 ± 0.3%. The yarn could withstand cyclic tensile deformation to 3, 4, and 5% strain, confirming its suitability for textile manufacturing. The robustness and utility of the *n*‐type yarn were illustrated by the fabrication of two out‐of‐plane thermoelectric textile devices: a thermoelectric button and a larger textile thermopile. PBFDO and PEDOT:PSS silk yarns were used to hand‐stitch a button onto a pile of wool fabrics, resulting in a thermocouple with a maximum output power of ≈20 nW and good air stability for at least one year. Additionally, PBFDO and PEDOT:PSS silk yarns were used to fabricate a larger thermoelectric textile device consisting of 8 *n*‐type and 8 *p*‐type legs. The open‐circuit voltage *V*_*oc*_of the device reached a value of up to 17 mV and a maximum output power *P*_max _= 0.67 µW for a Δ*T* = 70 K, which, e.g., would be sufficient to power sensors based on organic electrochemical transistors (OECTs) for sensing of electrophysiological signals.^[^ ^53^, ^54^ ^]^ Numerical simulations were in good agreement with the experimentally obtained device performance and indicated that *P*_max_ could be increased by minimizing the electrical contact resistance. Evidently, PBFDO coated multifilament silk yarn is a promising material for the realization of stable thermoelectric textile devices.

## 4 Experimental Section

### Materials

PEDOT:PSS (Clevios PH1000) was purchased from Heraeus Holding GmbH. PBFDO was synthesized according to the literature.^[^ ^47^ ^]^ 3,7‐dihydrobenzo[1,2‐*b*:4,5‐*b*′]difuran‐2,6‐dione (H‐BFDO), duroquinone and DMSO (purity 99.9%) were purchased from Sigma–Aldrich and used as received. H‐BFDO (1 eq, 150 mg) and duroquinone (1.5 eq, 194 mg) were dissolved in 10 mL DMSO under nitrogen, followed by heating at 100 °C for 1 h. The final mixture was dialyzed against DMSO using a dialysis bag with a cutoff molecular weight of 10 kg mol^−1^ (Viskase, USA) for 1–2 weeks (DMSO was changed every 3 days until it became colorless). The mixture in the dialysis bag was filtered with a 0.45 µm polytetrafluoroethylene filter to obtain the final PBFDO:DMSO ink.

### Sample Preparation and Yarn Coating

Thin films were spin coated (1500 rpm for 120 s and 3000 rpm for 10 s) onto cleaned glass slides (prewashed by sonication in acetone and then isopropanol) and then annealed on a hotplate at 50 °C for 1 h. Free‐standing films with a thickness of 7–18 µm were prepared by drop casting PBFDO:DMSO ink onto cleaned glass slides at 40 °C, followed by drying in a vacuum oven for 2 days at 40 °C, and finally removal from the substrate with a sharp blade. The thickness of thin films was determined with atomic force microscopy (AFM) in tapping mode using a Dimension 3000 instrument from Digital Instruments equipped with a standard silicon tip; the thickness of free‐standing films was measured with a digital caliper. Silk sewing thread was purchased from Aurora Silk. A prewash of silk yarn in a 1:50 (yarn:water) wash bath using 4 g dm^−3^ (with respect to water) silk detergent (Zenit) and 4 g dm^−3^ ammonia (25%, Merck Millipore) was performed. PBFDO coated silk yarn was prepared by immersion into PBFDO:DMSO ink for 10 to 20 min, followed by drying in an oven at 40 °C. Dip coating in PBFDO:DMSO ink was repeated twice (for the yarns used for the characterization and for sewing the thermopile) or three times (for the yarns used to fabricate the prototype), followed by a final drying step at 40 °C. Oxidation of PBFDO coated yarn was achieved by submerging yarn sections for 20 min in solutions of 0.1–3 mm Magic Blue (obtained from Sigma–Aldrich), dissolved in 2 mL dry and degassed (30 min N_2_) acetonitrile (obtained from Sigma–Aldrich). PEDOT:PSS coated yarns were prepared according to a previously reported roll‐to‐roll method.^[^ ^51^ ^]^

### Scanning Electron Microscopy

Samples for scanning electron microscopy (SEM) were freeze‐fractured in liquid nitrogen, and then sputtered with gold, were indicated. SEM imaging was done with a LEO Ultra 55 (Zeiss, Germany) at an acceleration voltage of 2–3 kV.

### Wide Angle X‐Ray Scattering (WAXS)

Wide‐angle X‐ray scattering was carried out in transmission mode on bulk samples with a Mat:Nordic instrument from SAXSLAB equipped with a Rigaku 003+ high‐brilliance microfocus Cu Kα radiation source (wavelength = 1.5406 Å) and a Pilatus 300 K detector placed at a distance of 124.6 mm from the sample.

### Electrical Characterization

The electrical resistance of thin films was measured by the four‐point probe method in vacuum from 80 to 400 K with 10 K steps using a semiconductor characterization system (Keithley 4200‐SCS). The temperature was controlled by a heater and liquid nitrogen. Four parallel line‐shaped Cr/Au electrodes were evaporated on glass substrates with a channel width of 12 mm and a channel length of 0.6 mm on top of which PBFDO films were spin coated. To determine the electrical conductivity of conducting yarns, samples were placed on a glass substrate, and contact points with a spacing of *L* = 10 mm were made on top using silver paint (fast drying silver suspension from Agar Scientific Ltd.). The resistance of each 10 mm long section was measured using a Keithley 2400 source‐measure unit in 2‐point configuration. The electrical contact resistance *R*_*c*, *yarn*_ was estimated through a transmission line method, by plotting the electrical resistance of yarn sections as a function of section length followed by extrapolation of *R*_*c*, yarn_ at *L* = 0. The measured resistance *R_m_* was then corrected according to *R*  = *R_m_*  − *R*_*c*, yarn_. The effective electrical conductivity was calculated according to σ_eff_ =  *L*/(*A* · *R*) where *L* is the length of the characterized yarn section, i.e., 10 mm, and *A*  =  π*D* ^2^/4 is the yarn cross‐section area determined by assuming an homogenous circular area and measuring the yarn diameter *D* with an Axio optical microscope from Zeiss. The Seebeck coefficient at 300 K was measured with an SB1000 instrument from MMR Technologies equipped with a K2000 temperature controller using a thermal load of 1–2 K and a constantan wire as internal reference. 5 mm‐long yarn segments were mounted on the SB1000 sample stage with silver paint.

### Mechanical Characterization

Tensile deformation of free‐standing films and yarn sections was performed using a DMA Q800 instrument from TA Instruments in controlled force mode at different temperatures. A force rate of 0.01 N min^−1^ for polymer films and 0.1 N min^−1^ for yarns were used and the preload force was 0.005 N. Cyclic tensile deformation of conducting yarn was performed using the same DMA Q800 instrument by repeatedly applying 3, 4, or 5% maximum strain for 12 s followed by a 60 s release. The electrical resistance during cyclic deformation was monitored with a Keysight U1253B multimeter. Dynamic mechanical thermal analysis (DMTA) was performed using a DMA Q800 in tensile mode at a frequency of 1 Hz while ramping the temperature from −80 to 200 °C at a rate of 3 °C min^−1^. A dynamic strain with a maximum value of 0.1% and a preload force of 0.001–0.005 N were used.

### Machine Washing

Machine washing was carried out using a commercial washing machine (Daewoong Morning Calm) with 30 mL of commercial detergent (Spark, Aekyung Co. Ltd.) in 3 L of water. The detergent was reported to contain linear alkylbenzenesulfonates, α‐olefins, zeolite, fatty acids, etc. Several samples were prepared, each comprising three segments of ≈5 cm long *n*‐type yarn that were hand‐stitched onto a piece of felted wool fabric. Each felt was put into a mesh laundry bag and then washed 1 to 7 times in the washing machine with 3 L of water. Each washing cycle was performed for 15 min at 20 °C. After each washing cycle, the electrical resistance of the three yarns stitched on the felt was measured using a multimeter (Fluke) in 2‐point configuration.

### Textile Thermoelectric Button and Generator

A button (circular shape with a diameter of 15 mm) was sewn onto three layers of felted wool fabric from Harry Hedgren AB (Wadmal, 3.2 g dm^−2^, ≈1 mm thick) by hand stitching the conducting yarns with a sewing needle to form a thermopile comprising one *n*‐type and one *p*‐type leg. After stitching, silver paint (fast drying silver suspension from Agar Scientific Ltd) was applied to both the bottom and top surfaces to establish electrical connections between the legs that also served as contact pads for device characterization.

PBFDO and PEDOT:PSS‐coated yarns were hand stitched onto six layers of felted wool fabric from Harry Hedgren AB (Wadmal, 3.2 g dm^−2^, ≈1 mm thick) using a sewing needle. The sewn yarns formed an out‐of‐plane thermoelectric generator comprising 8 *n*‐type and 8 *p*‐type legs. Each leg was characterized by an area of 8–10 mm times 8–10 mm and a length *L_l_*, i.e., the thickness of the felted wool layers, of 8 mm. After stitching, silver paint (fast drying silver suspension from Agar Scientific Ltd) was applied with a circular (diameter ≈ 8 mm) rubber stamp to both the bottom and top surfaces to improve the electrical contact among the conducting yarns of each leg and to establish electrical connections between the legs.

The thermoelectric button and the textile thermoelectric generator were characterized by placing them on top of a variable temperature hot plate (HP60, Torrey Pines Scientific Inc.). K‐type thermocouples (Omega Engineering) were placed on the top and at the bottom of the devices to record the surface temperatures using a cDAQ 9174 instrument from National Instruments with an internal temperature reference. A cooling element was placed on top of the devices (separated by a glass slide to avoid direct contact) to maintain a constant cold temperature and to keep the device in place by providing a weight of ≈0.75 kg. The generated voltage was measured with a Keithley 2400 source measure unit. Furthermore, the instrument was used as a variable load by drawing current from the thermoelectric generator to determine the maximum output power.

### Modeling and Numerical Simulation

A 3D model of the textile thermopile was constructed in COMSOL Multiphysics. The development of the simulation model was based on the following assumptions: 1) only heat conduction was considered, while heat transfer via convection and radiation was negligible; 2) the influence of Joule heating, the Peltier effect, and the Thomson effect was negligible.

The geometrical and material parameters were selected based on the experimental results (Table 2; Figure S19a, Supporting Information). The side length of the whole model and each leg was 55 and 8 mm, respectively, in line with the actual textile thermopiles. The spacing between each leg was set to be 3 mm. The length of each thermoelectric leg *L_l_* was varied from 2 to 30 mm for investigating the optimal length *L_l_* leading to the *P*_max_. Electrodes were constructed for electrically connecting the legs (Figure S19b, Supporting Information). The electrical resistance and thermal resistance of the electrodes were sufficiently small (at least 1000 times smaller than the corresponding contact resistance) to be neglected. The introduction of electrical and thermal contact resistance in the model was achieved by setting an electrical and thermal contact node between the legs and the electrodes.

A 1400 mm long coated yarn was used to fabricate each leg, which corresponds to a thread count *N_t_* of around 90. The cross‐sectional area of coated yarn *A_y_* per leg was given by:

(5) $$A_{y}=\frac{N_{t}\pi D^{2}}{4}\approx 2.83\;mm^{2}\;$$

where *D* is the yarn diameter.

The internal electrical resistance *R*_int,l_ of *p*‐type and *n*‐type legs were calculated to be 2.19 and 2.16 Ω, respectively, using the equation below:

(6) $$R_{\mathrm{int},l}=\frac{L_{l}}{A_{y}\sigma_{\mathrm{eff}}}\;$$

where *L_l_* is the length of the leg and σ_eff_ is the bulk conductivity of the conducting yarns.

The total internal electrical resistance of the whole textile device was measured to be 119.0 Ω at room temperature and 107.9 Ω at 90 °C, i.e., the set temperature of the hot plate to obtain a Δ*T* = 70 K (Figure S16, Supporting Information). Assuming that *R*_int,l_ of the *n*‐type yarn was constant up to 100 °C (see Figure S20, Supporting Information), and that *p*‐type yarn shows a similar behavior, a change in the electrical contact resistance *R_c_* per thermocouple with increasing temperature is estimated, ranging from 10.5 Ω at room temperature to 9.1 Ω at 90 °C.

Cubic legs with an effective electrical and thermal conductivity were utilized to represent the legs made of felted wool fabric and coated yarns. The effective electrical conductivity of the *p*‐type or *n*‐type legs was given by:

(7) $$\sigma_{l}=\frac{L_{l}}{A_{l}\;R_{\mathrm{int},l}}\;$$

where *A_l_* is the cross‐sectional area of the legs.

Regarding the heat conduction across the legs, it was assumed that the thermal resistances of coated yarns *K_y_* and felted wool fabric *K_w_* were coupled in parallel. *K_y_* is given by $K_{y}=\frac{L_{l}}{\lambda_{y}A_{y}}\;$, while $K_{w}=\frac{L_{l}}{\lambda_{w}(A_{l}-A_{y)}}$, where λ_*y*_ and λ_*w*_ are the thermal conductivity of the coated yarn and felted wool fabric, respectively. *p*‐type yarn was characterized by a λ_*y*_ = 0.18 W m^−1^ K^−1^, as previously reported,^[^ ^19^ ^]^ and the same value for the *n*‐type yarn was assumed. The thermal conductivity of felted wool λ_*w*_ used in the simulation was 0.056 W m^−1^ K^−1^, as previously reported.^[^ ^19^ ^]^ The total thermal resistance *K* of each leg was given by:

(8) $$\frac{1}{K}=\frac{1}{K_{y}}+\frac{1}{K_{w}}\;$$

The effective thermal conductivity of the cubic legs was calculated by $\lambda_{l}=\frac{L_{l}}{KA_{l}}\;$.

## Conflict of Interest

S.F. is a co‐founder and chief scientific officer of the company n‐ink. All other authors declare no conflict of interests.

## Supporting information

Supporting Information

## Data Availability

The data that support the findings of this study are openly available in Zenodo at https://zenodo.org/doi/10.5281/zenodo.10926630.
