# Supplementary material for: Poly(benzodifurandione) Coated Silk Yarn for Thermoelectric Textiles
Source: Adv Sci (Weinh). 2024 Aug 5;11(38):2406770. doi: 10.1002/advs.202406770 (PMC11481370; doi:10.1002/advs.202406770)
Supplement: Supplementary file 1 — Supporting Information [file ADVS-11-2406770-s001.docx]

Supporting Information

**Poly(benzodifurandione) Coated Silk Yarn for Thermoelectric Textiles**

Mariavittoria Craighero^1#^, Qifan Li^2#^, Zijin Zeng^1,3^, Chunghyeon Choi^4^, Youngseok Kim^1^, Hyungsub Yoon^4^, Tiefeng Liu^2^, Przemyslaw Sowinski^1^, Shuichi Haraguchi^1^, Byungil Hwang^5^, Besira Mihiretie^3^, Simone Fabiano^2^*, Christian Müller^1^*

^1^Department of Chemistry and Chemical Engineering, Chalmers University of Technology, 41296 Göteborg, Sweden

^2^Laboratory of Organic Electronics, Department of Science and Technology, Linköping University, 60174 Norrköping, Sweden

^3^Hot Disk AB, Sven Hultins gatan 9A, 41258 Göteborg, Sweden

^4^Department of Intelligent Semiconductor Engineering, Chung-Ang University, 06974 Seoul, Republic of Korea

^5^School of Integrative Engineering, Chung-Ang University, 06974 Seoul, Republic of Korea

^#^these authors contributed equally

* [simone.fabiano@liu.se](mailto:simone.fabiano@liu.se); [christian.muller@chalmers.se](mailto:christian.muller@chalmers.se)

**
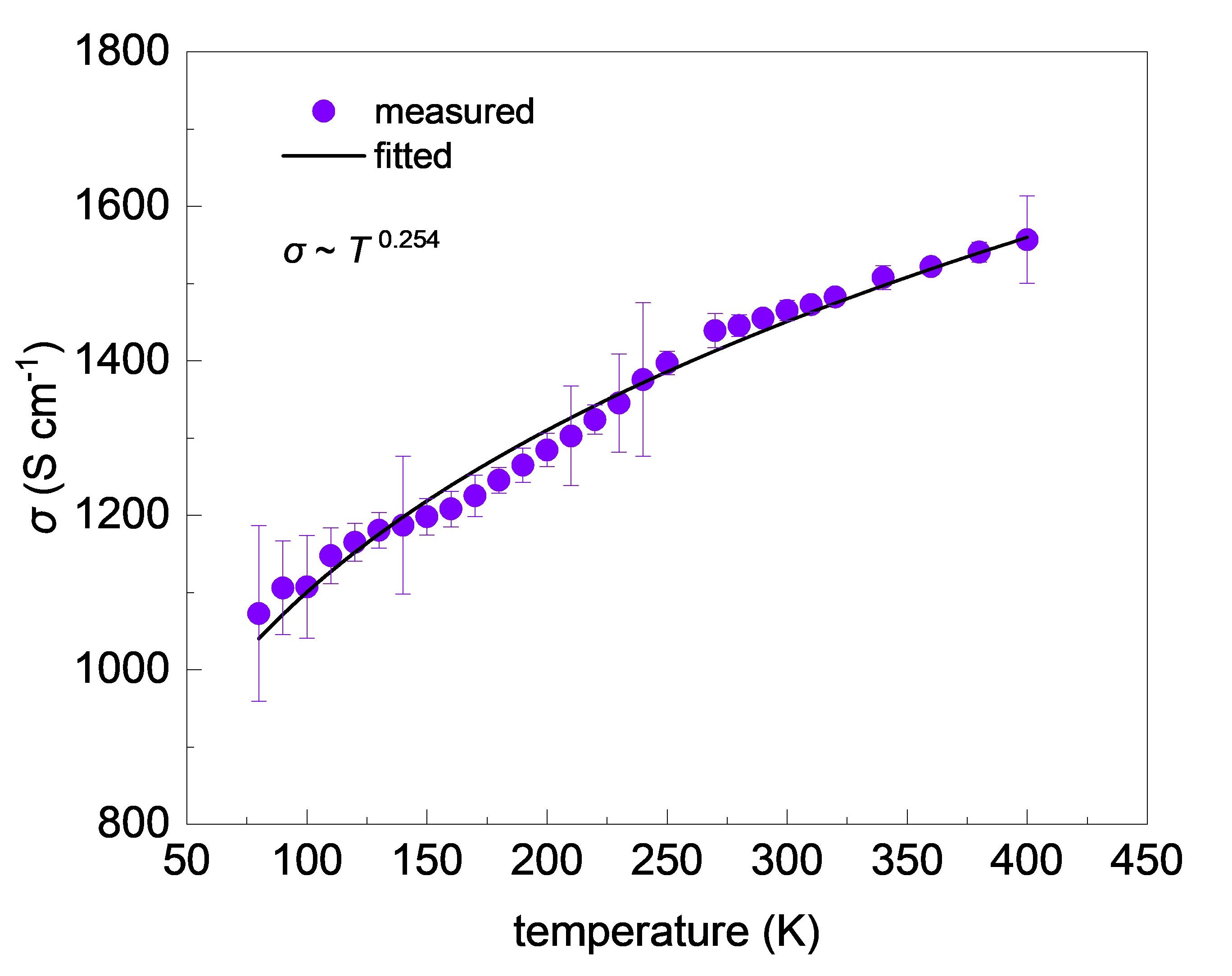
**

**Figure S1.** Electrical conductivity $\sigma$ of spin-coated films as a function of temperature.

**
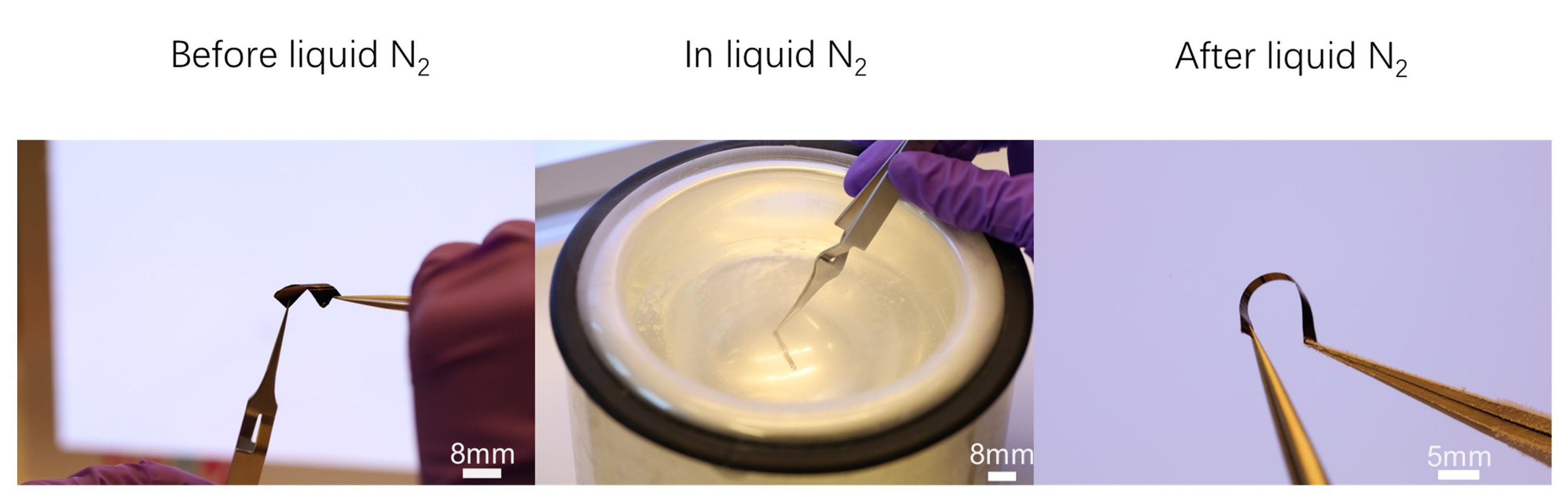
**

**Figure S2.** Photographs of PBFDO bent films before, during and after being immersed in liquid nitrogen for a few minutes.


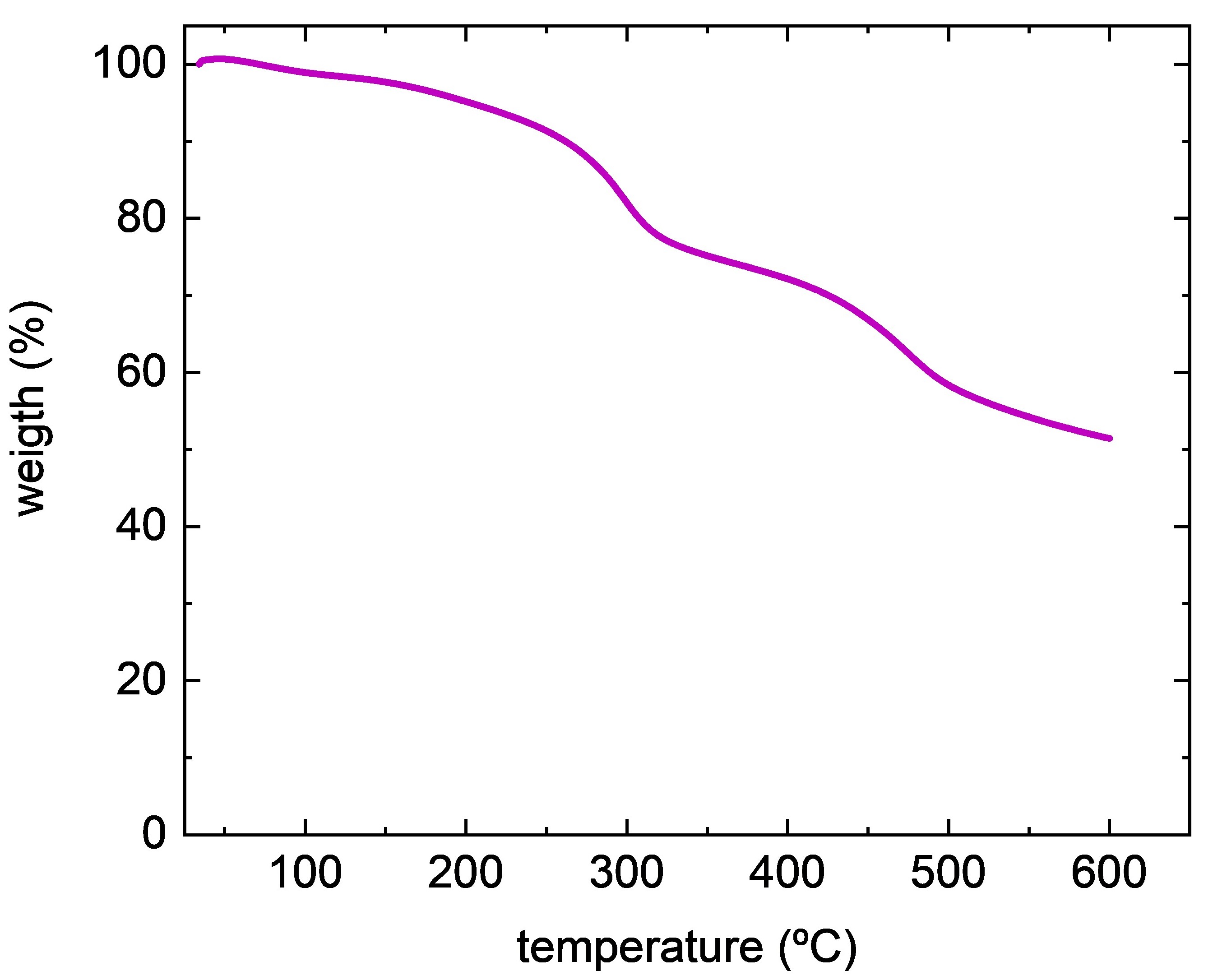


**Figure S3.** Thermogravimetric analysis (TGA) of PBFDO carried out under nitrogen at a scan rate of 10 °C min^−1^ using a Mettler Toledo TGA/DSC 3+ instrument.

**
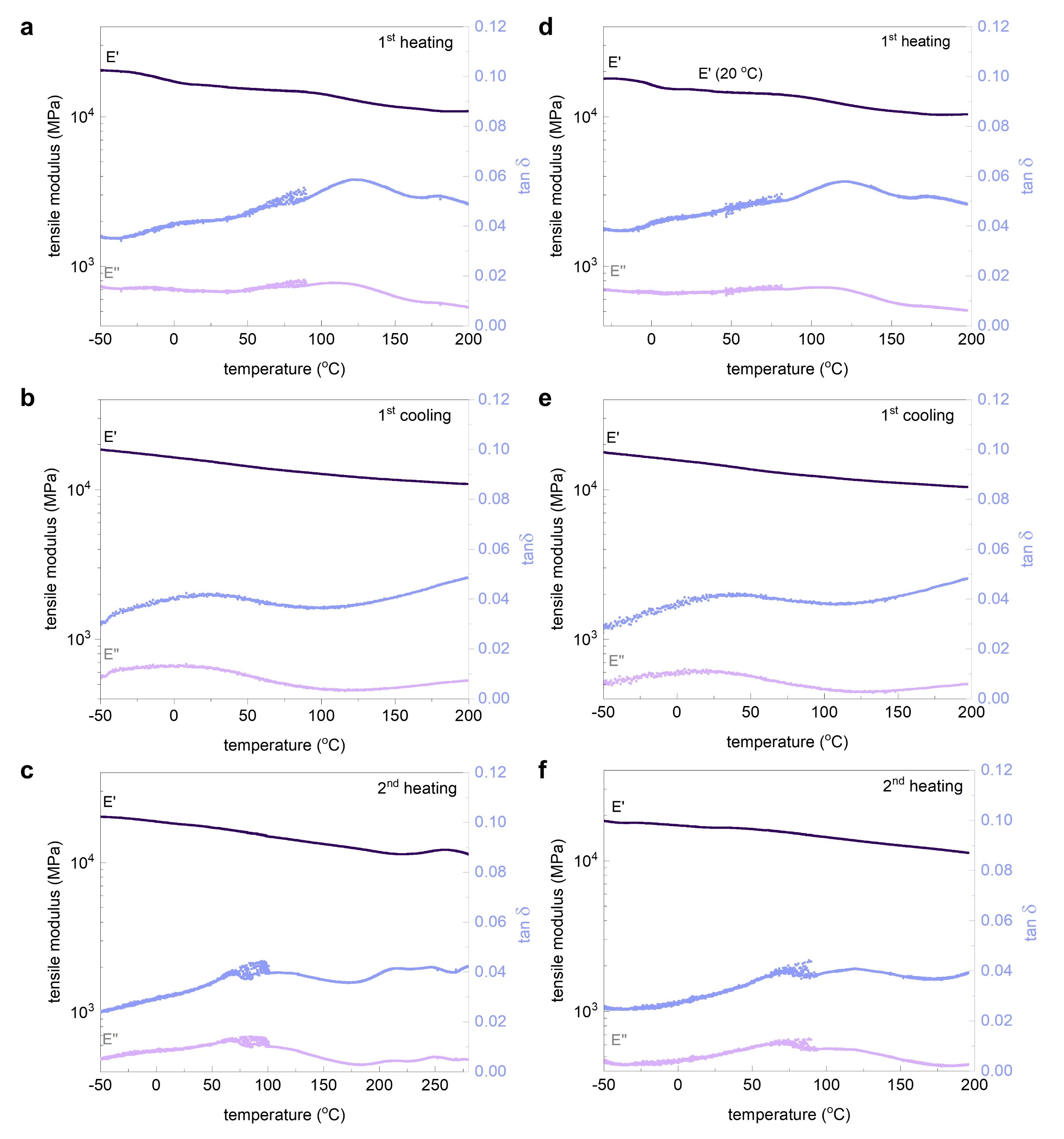
**

**Figure S4.** DMTA thermographs of two free-standing films (a-c, d-f) showing the storage modulus $E^{'}$, loss modulus $E^{''}$ and loss tangent $\tan\delta$ during the first heating (a, d), first cooling (b, e) and second heating (c, f) steps.


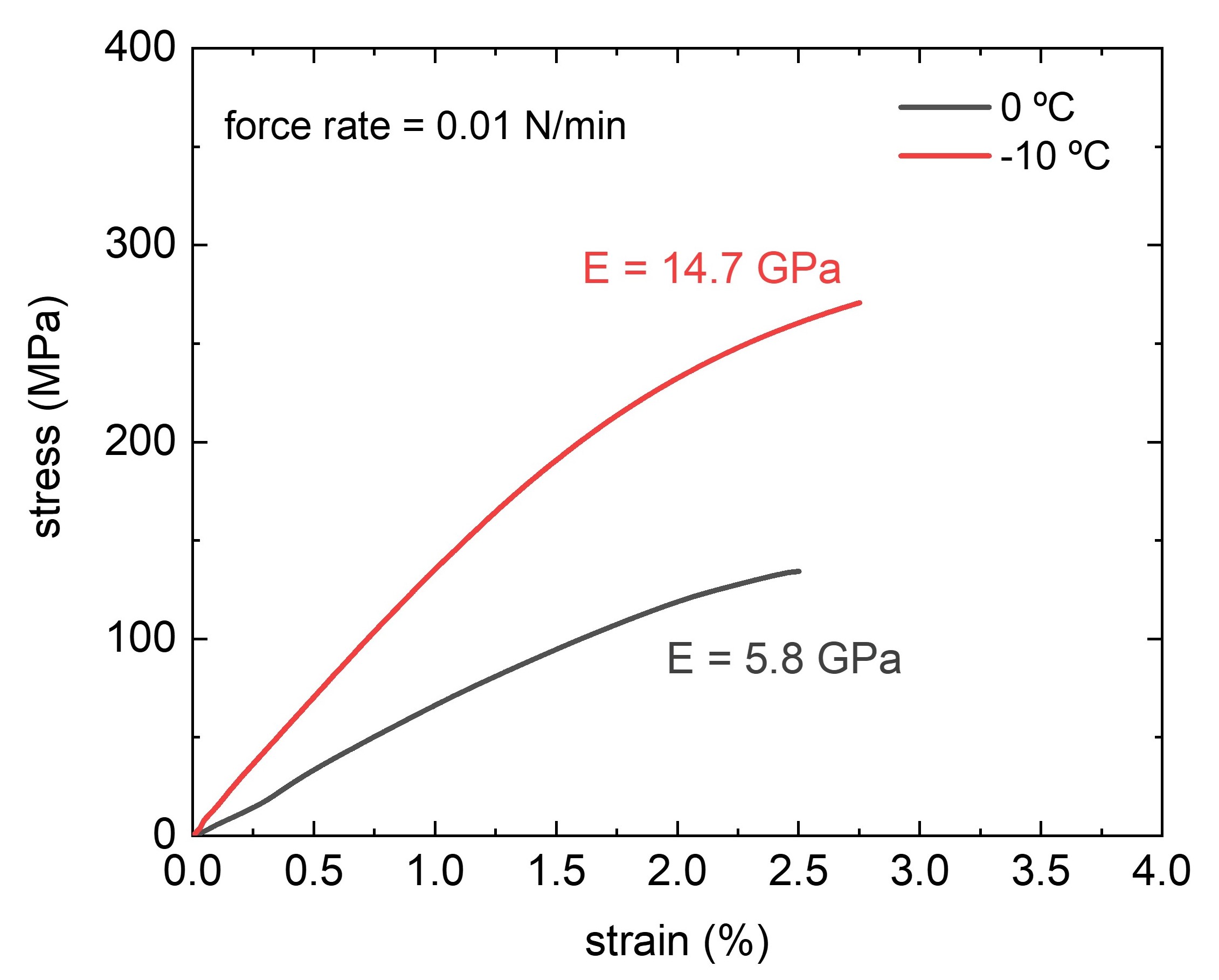


**Figure S5.** Stress-strain response of free-standing films at different temperatures measured by tensile deformation.

**
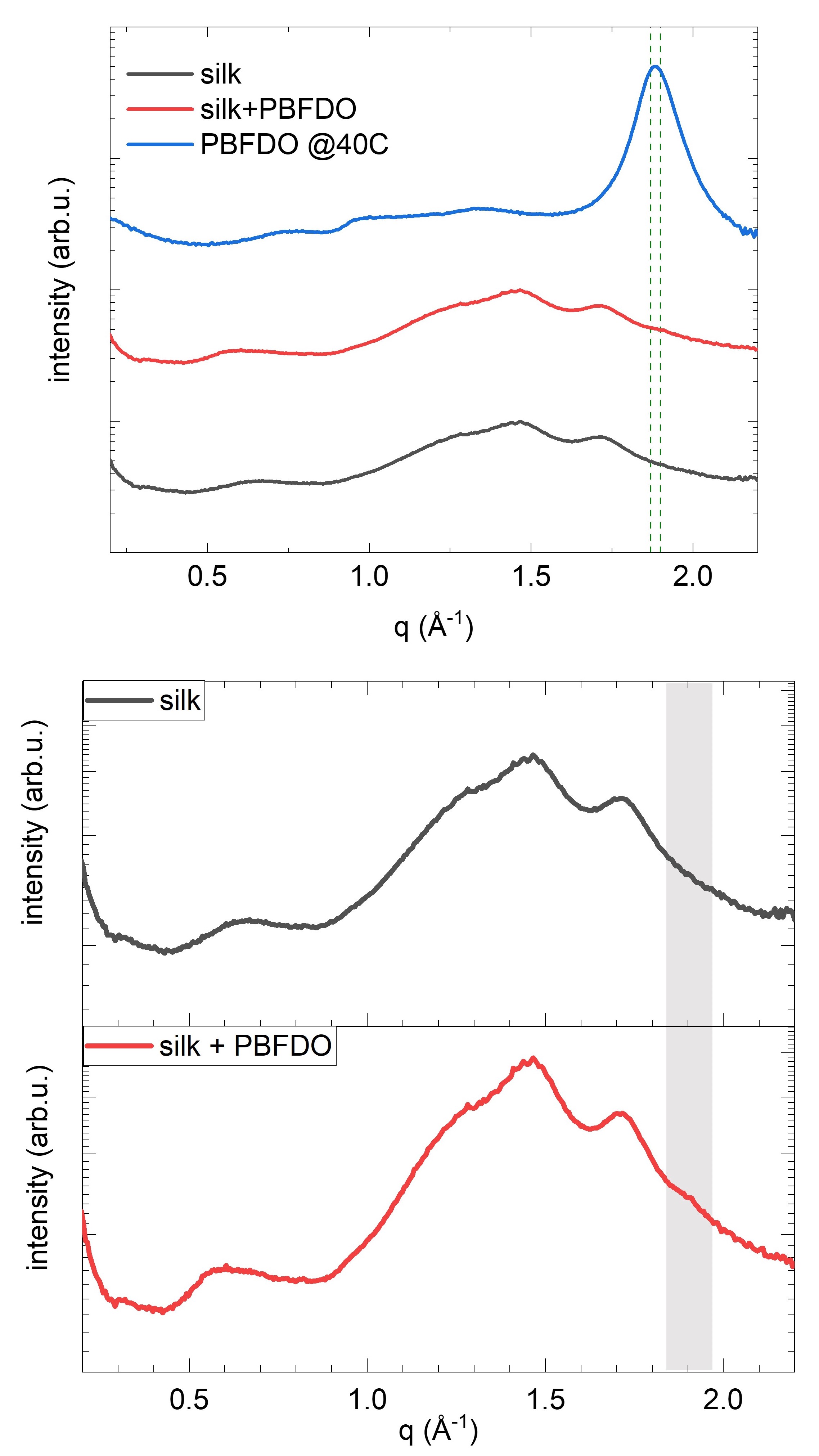
**

**Figure S6.** Transmission WAXS of neat and PBFDO-coated silk yarns.

**
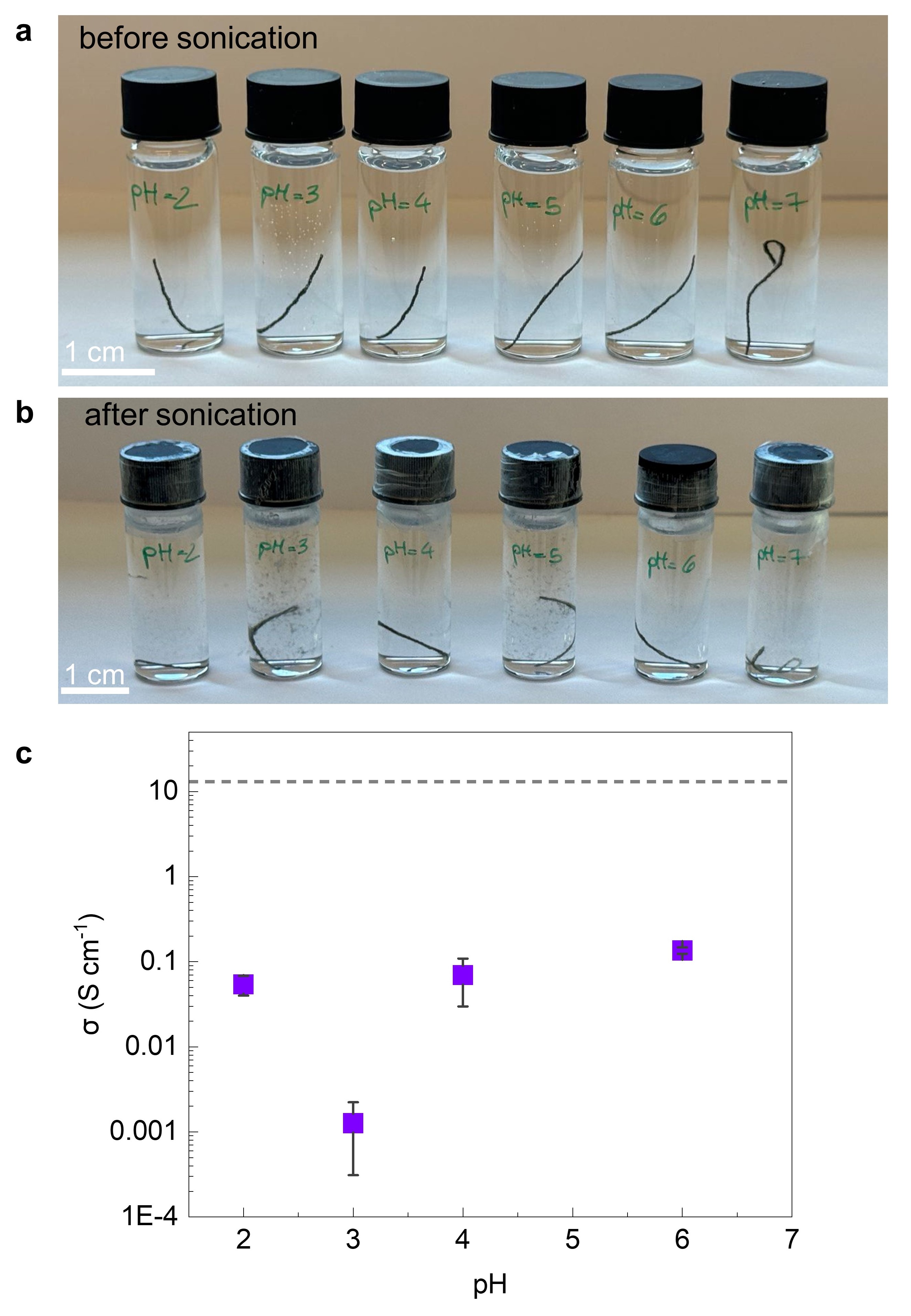
**

**Figure S7.** Photograph of PBFDO coated yarns suspended in water with a pH ranging from 2 to 7: before (a) and after (b) sonication for 20 minutes; (c) electrical conductivity of the conductive yarns after sonication (dashed line represents the electrical conductivity value of the yarns before the sonication).

**
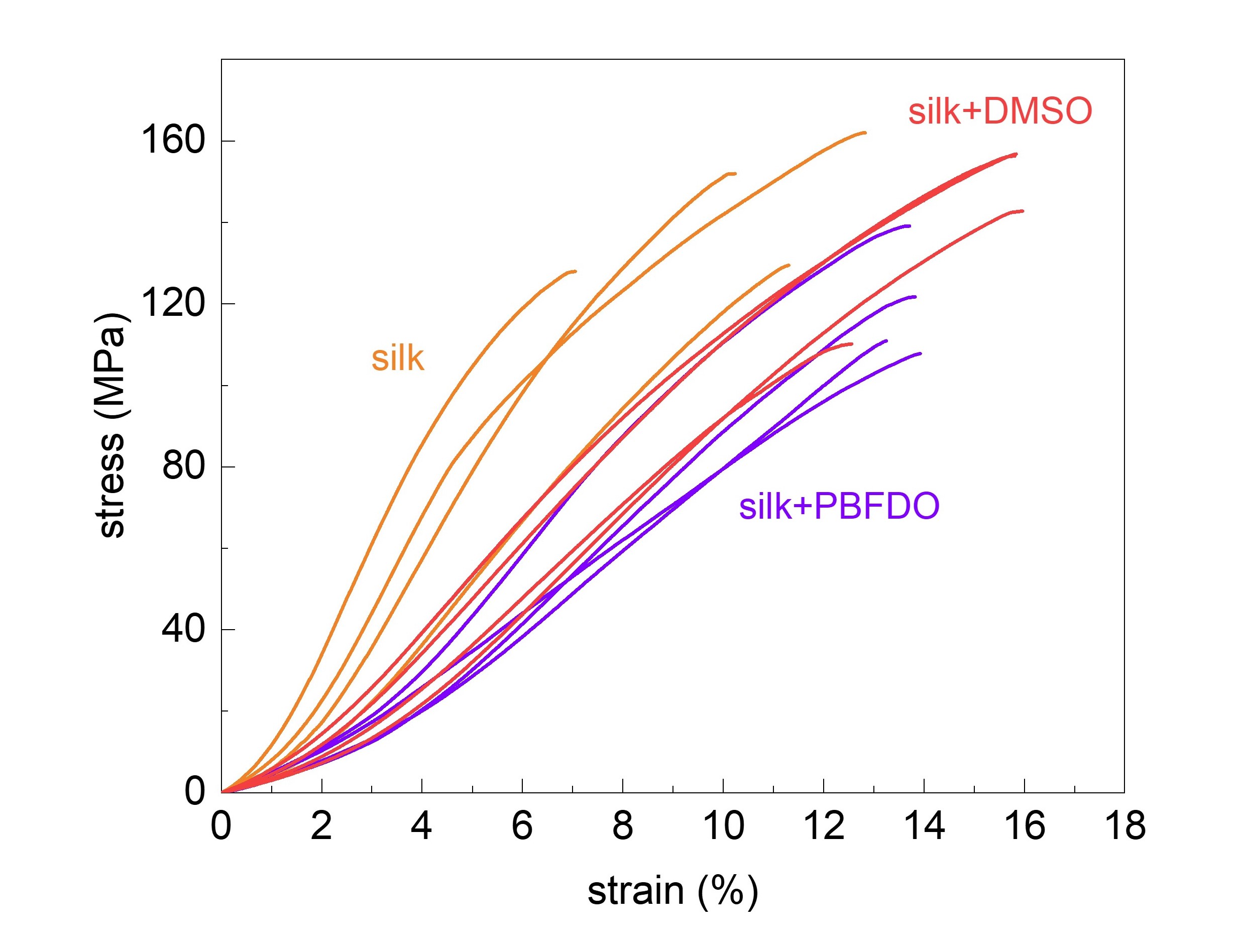
**

**Figure S8.** Stress-strain response of silk yarns: neat (orange), treated with DMSO (red), coated with PBFDO:DMSO ink (purple).

**Table S1.** Mechanical properties of coated silk yarns. Young’s modulus $E$ and strain at break $\varepsilon_{break}$. Values represent the mean and standard deviation of measurements of 5 samples.

| yarn | $E$(GPa) | $\varepsilon_{break}$(%) |
| --- | --- | --- |
| silk | 1.7 ± 0.5 | 10 ± 2.5 |
| silk treated with DMSO | 0.8 ± 0.2 | 15 ± 1.8 |
| PBFDO:DMSO coated silk | 0.6 ± 0.1 | 14 ± 0.3 |

**
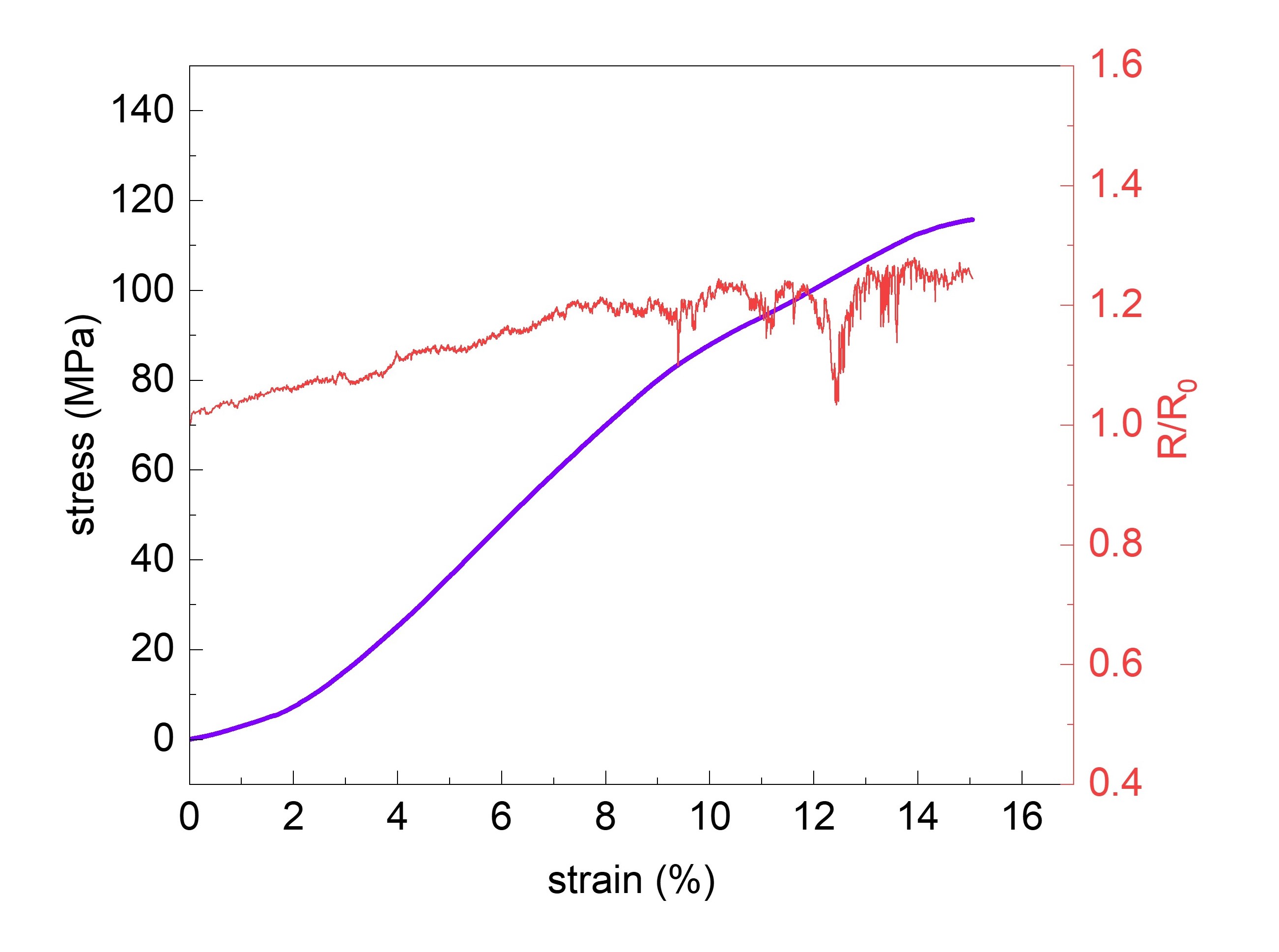
**

**Figure S9.** Stress-strain curve of the PBFDO coated silk yarn (left) and in-situ recorded change in electrical resistance $R/R_{0}$ where $R_{0}$ is the resistance of the yarn prior to the tensile test.

**
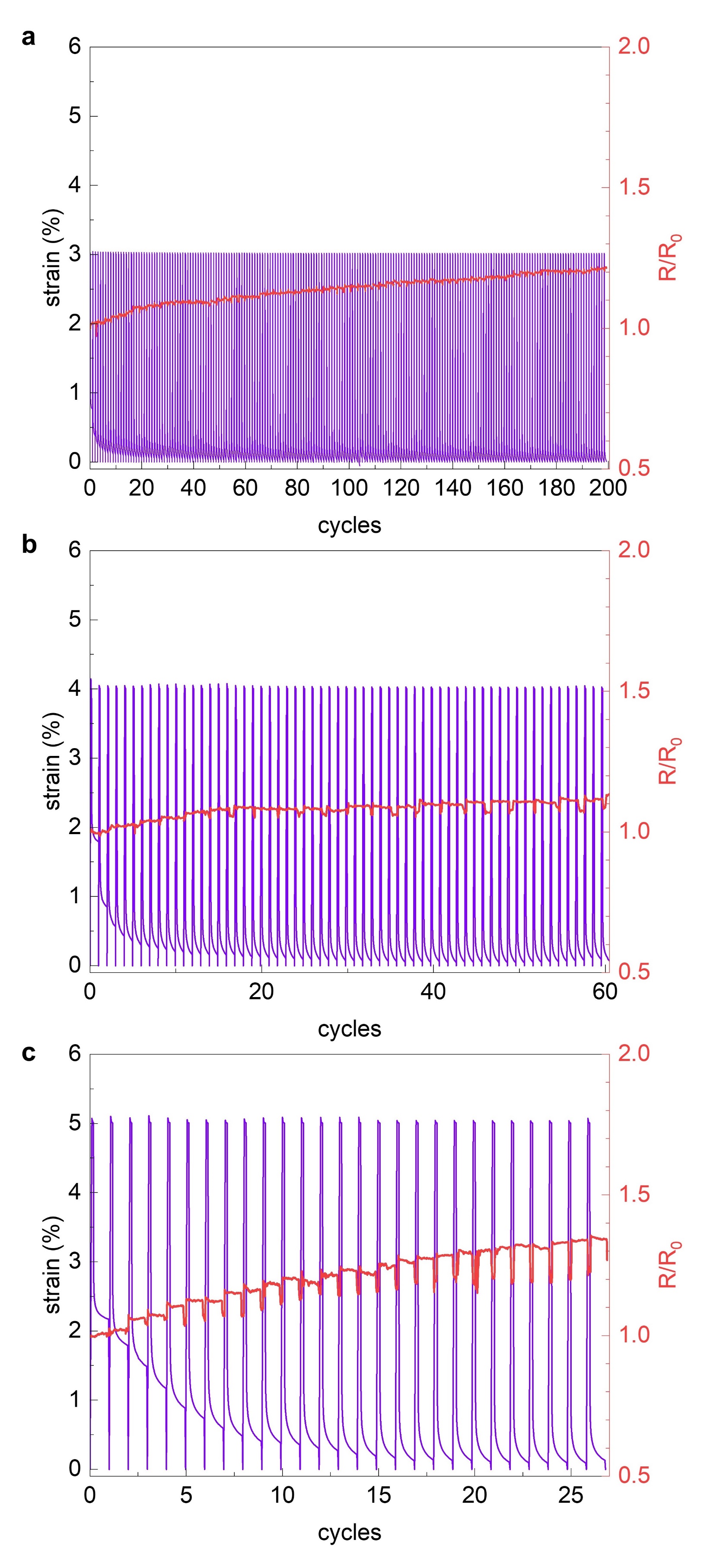
**

**Figure S10.** Strain during cyclic tensile deformation of the PBFDO coated silk yarn repeatedly stretched to 3 (a), 4 (b) and 5 % (c) then released for 60 s (purple line) together with the in-situ recorded change in electrical resistance (red line).


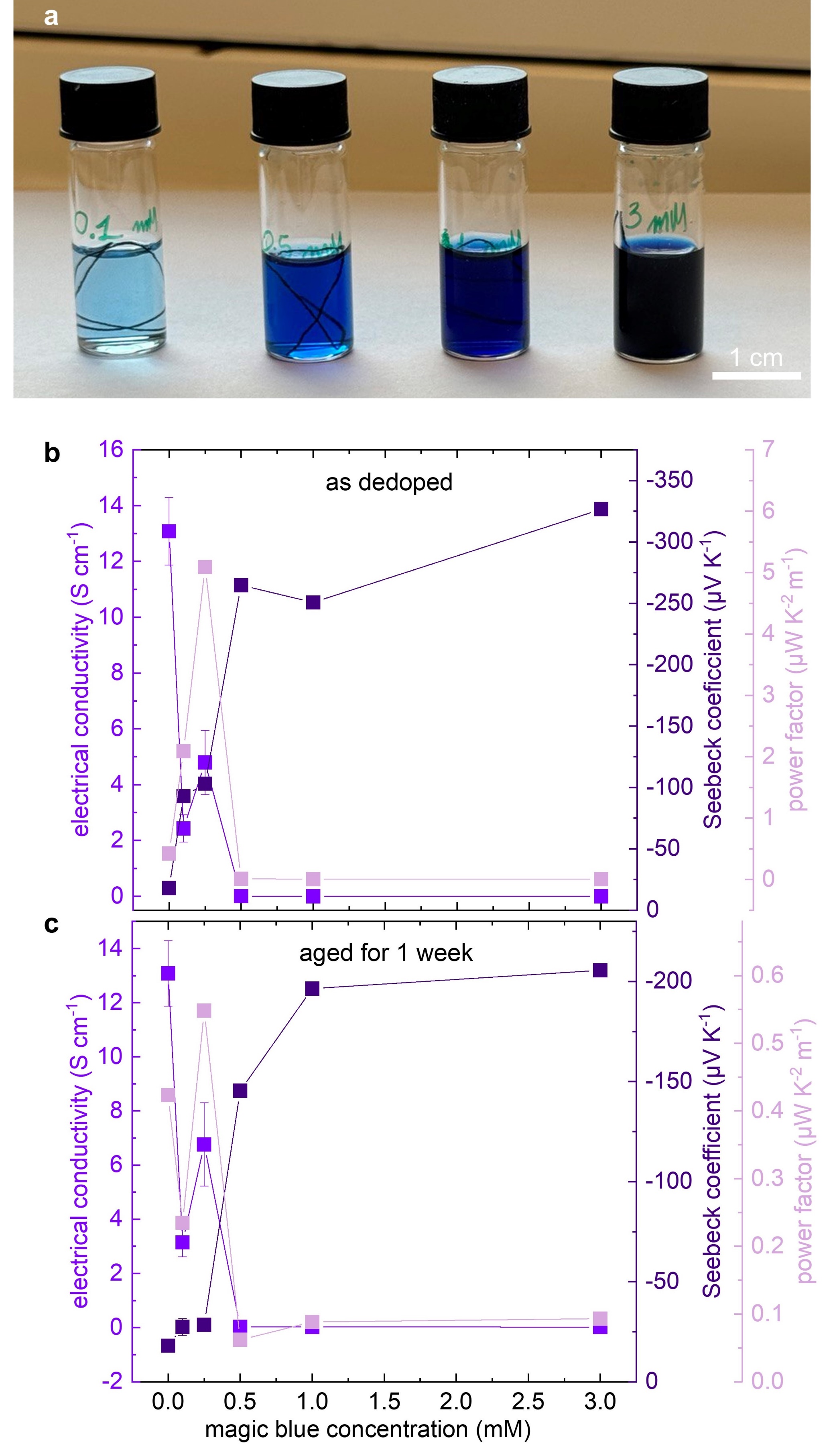


**Figure S11.** Oxidation of PBFDO coated yarns by Magic Blue. (a) Photograph of PBFDO coated yarns submerged into Magic Blue solutions with different concentrations; electrical conductivity (purple), Seebeck coefficient (dark purple) and power factor (pink) of PBFDO coated yarns as a function of Magic Blue concentration measured (b) as-dedoped and (c) aged for one week at ambient conditions.


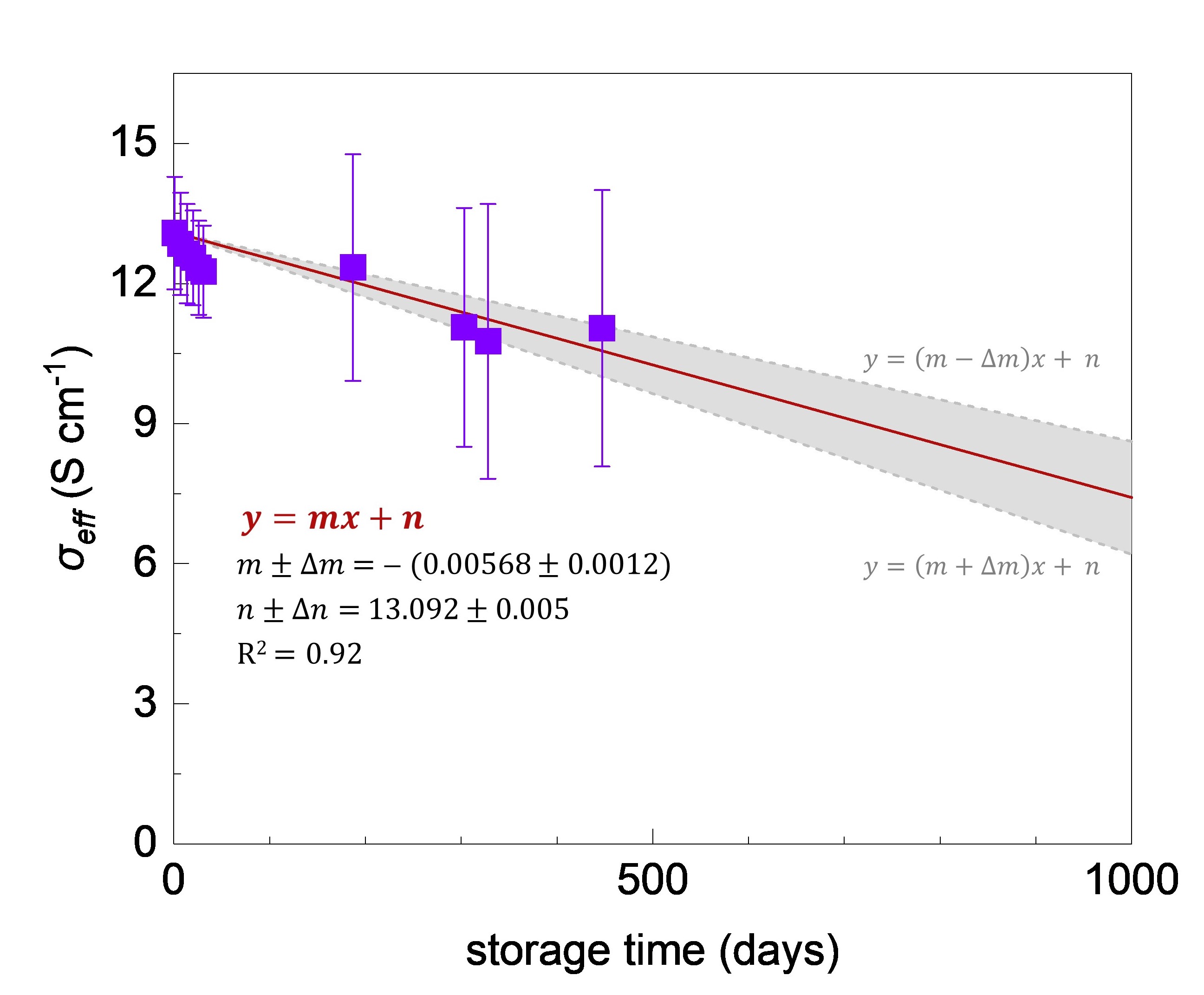


**Figure S12.** Effective electrical conductivity $\sigma_{eff}$ of PBFDO coated yarn (symbols) as a function of aging time at ambient conditions and linear fits with statistical results (grey lines: upper and lower boundaries calculated using $y=\left( m\pm\Delta m \right)x+n$, respectively; red line: mean fit) from which the half-life time was estimated.

**
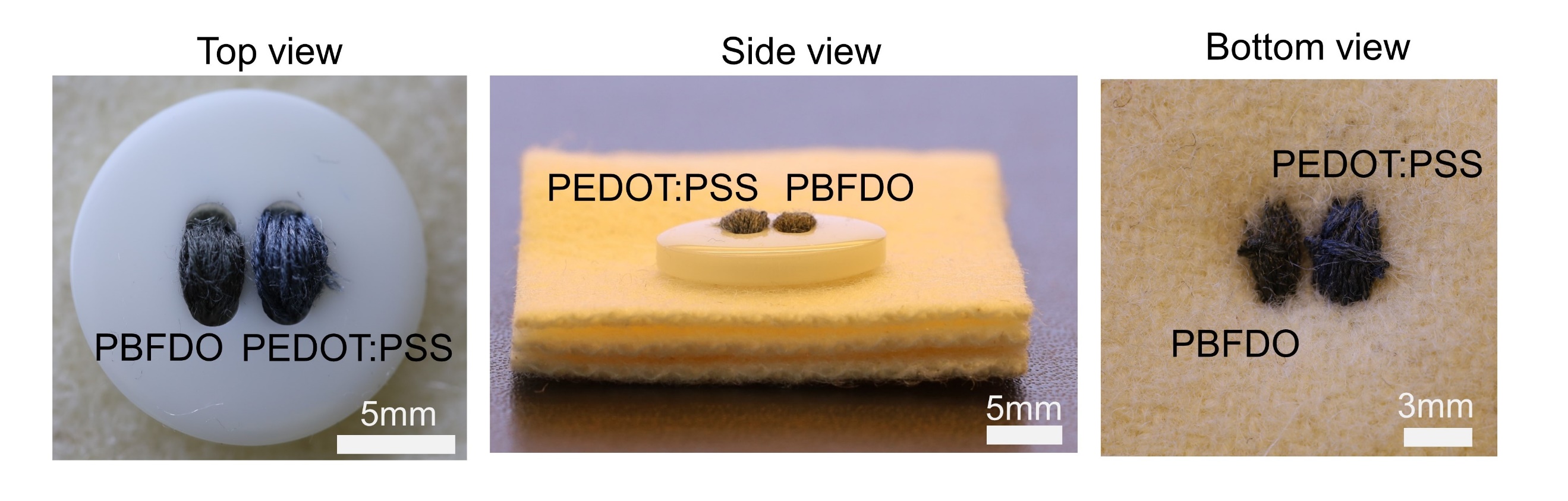
**

**Figure S13.** Photographs of the top, side and bottom view of the thermoelectric button.

**
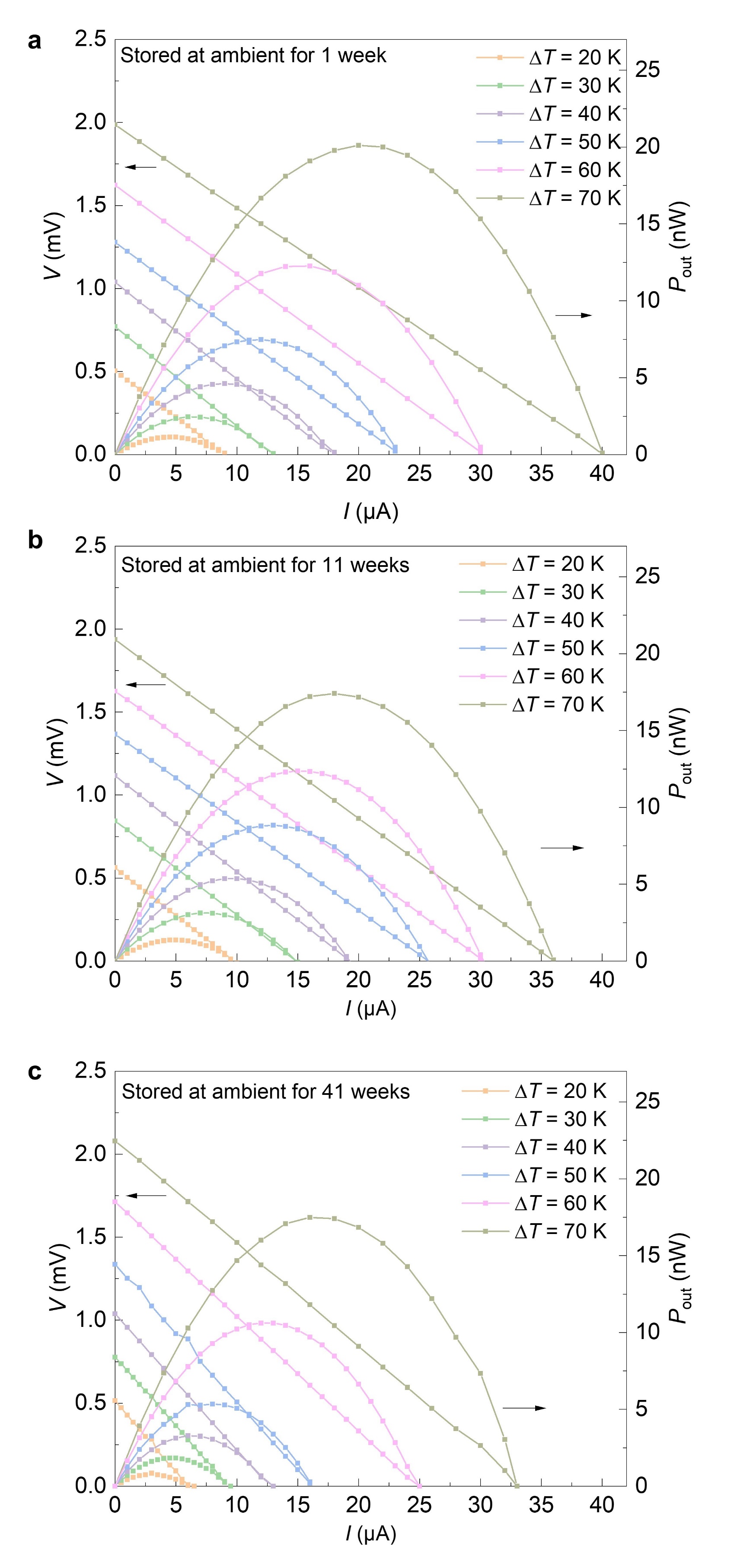
**

**Figure S14.** Performance of thermoelectric button stored at ambient conditions for (a) 1, (b) 11, (c) 41 weeks. Voltage $V$ (left) and output power $P_{out}$ (right) of the thermoelectric button as a function of current $I$ for different temperature differences $\Delta T=T_{hot}-T_{cold}$ where $T_{hot}$ and $T_{cold}$ are the temperatures of the hot plate and cooler measured with a pair of thermocouples.


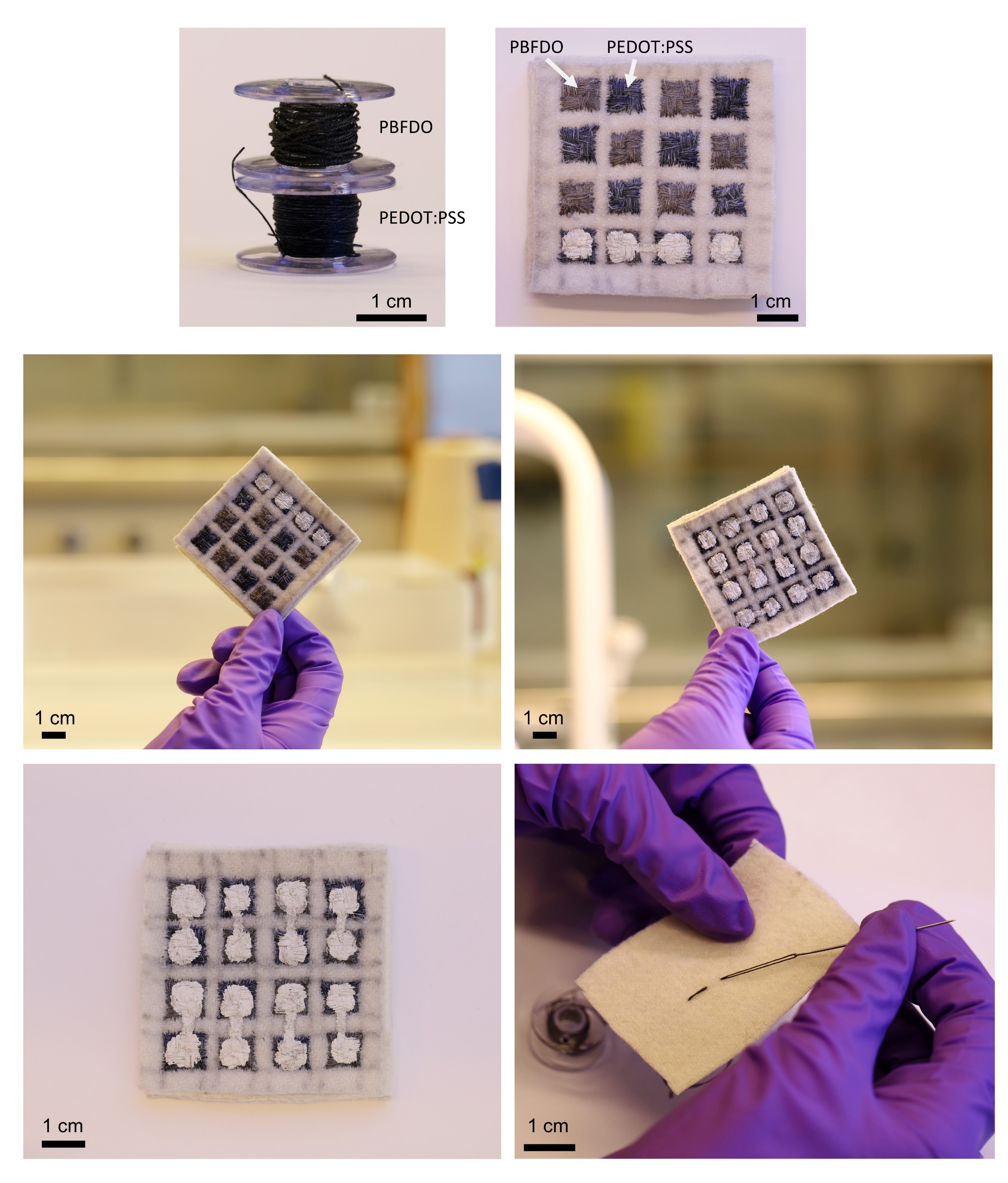


**Figure S15.** Photographs of PBFDO and PEDOT:PSS coated yarns and the thermoelectric generator.


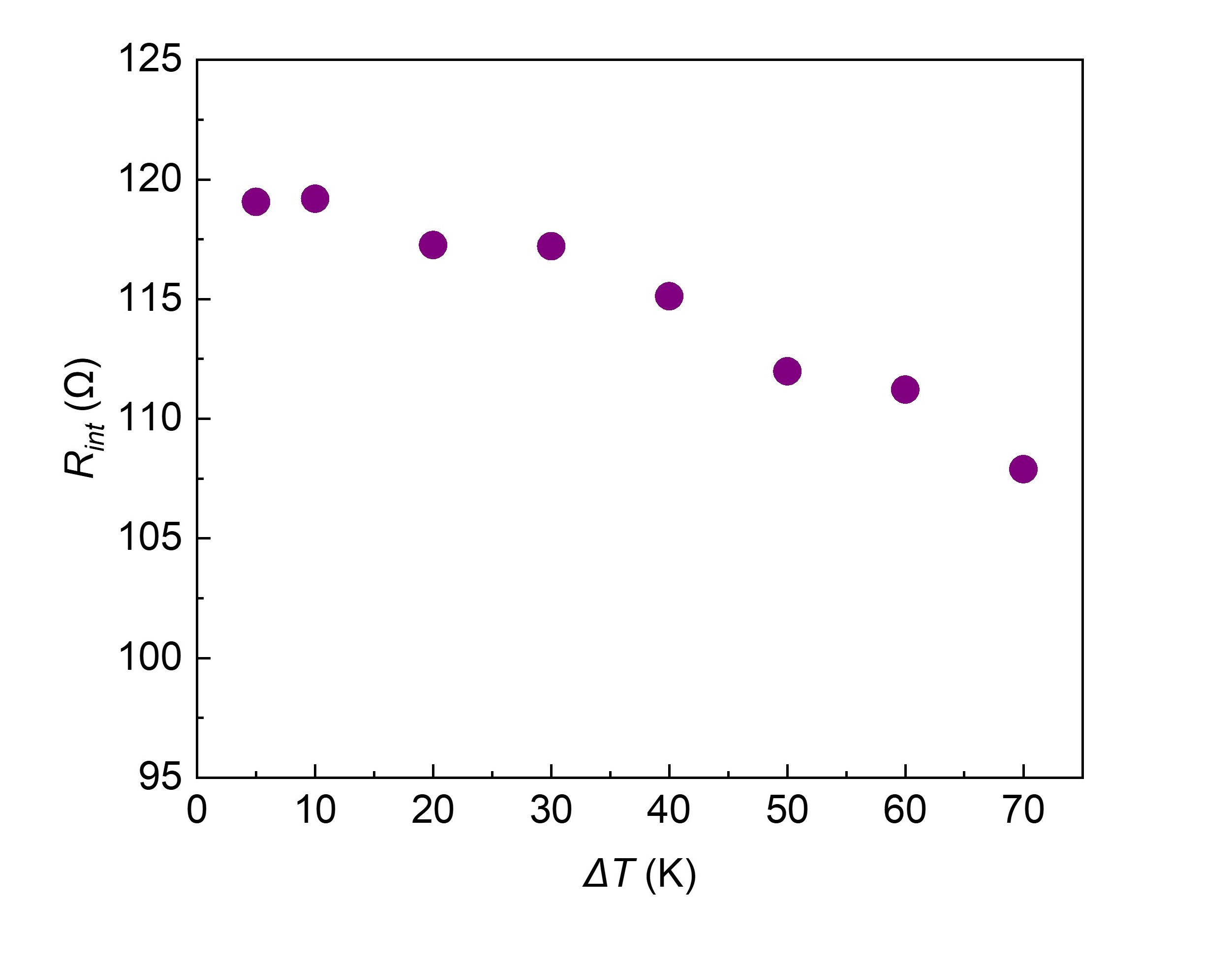


**Figure S16.** Internal electrical resistance $R_{int}$ of the thermoelectric generator as a function of the temperature gradient across the device.


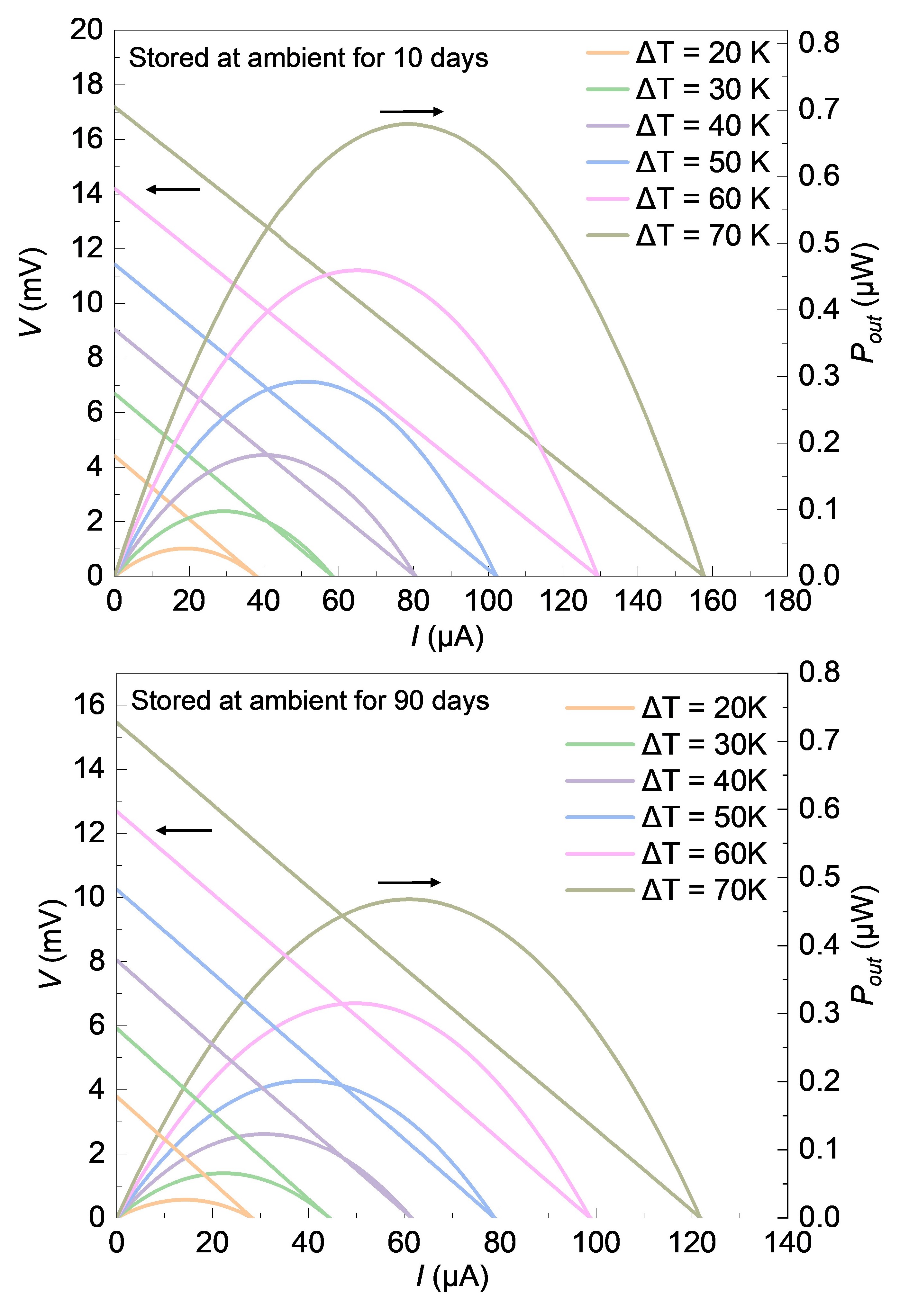


**Figure S17.** Performance of the larger thermoelectric generator stored at ambient conditions for 10 and 90 days. Voltage $V$ (left) and output power $P_{out}$ (right) of the thermoelectric device as a function of current $I$ for different temperature differences $\Delta T=T_{hot}-T_{cold}$ where $T_{hot}$ and $T_{cold}$ are the temperatures of the hot plate and cooler measured with a pair of thermocouples.


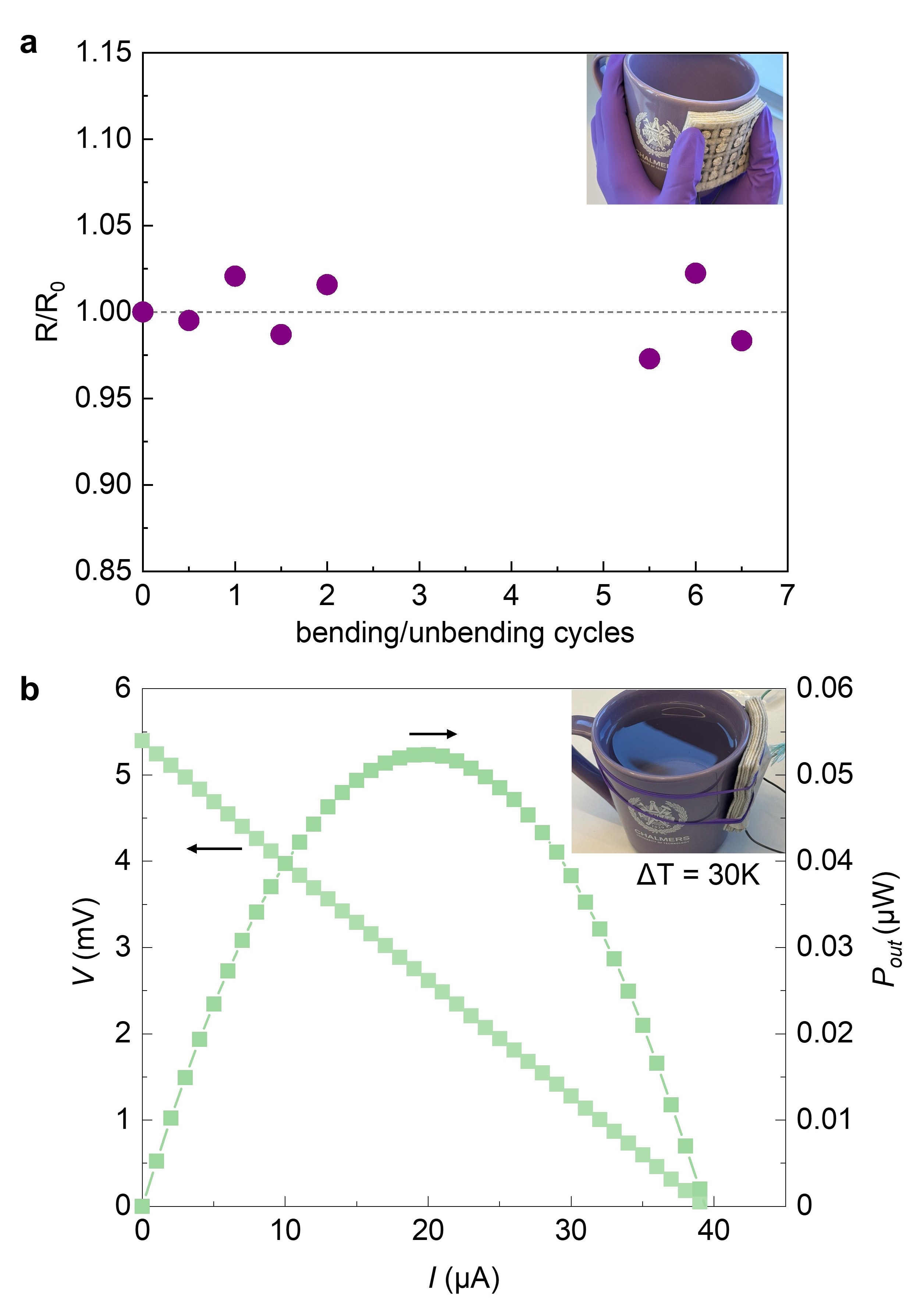


**Figure S18.** (a) Internal relative electrical resistance of the larger thermoelectric generator during bending-unbending cycles (inset photograph of the bent thermoelectric generator around a coffee mug); and (b) voltage $V$ (left) and output power $P_{out}$ (right) of the bent thermoelectric device as a function of current $I$ at temperature difference $\Delta T=T_{hot}-T_{cold}=30K$, where $T_{hot}$ and $T_{cold}$ are the temperatures of the warm wall of the mug filled with hot water and room temperature measured with a pair of thermocouples (the inset is a photograph of the thermoelectric generator bent around a coffee mug filled with hot water).


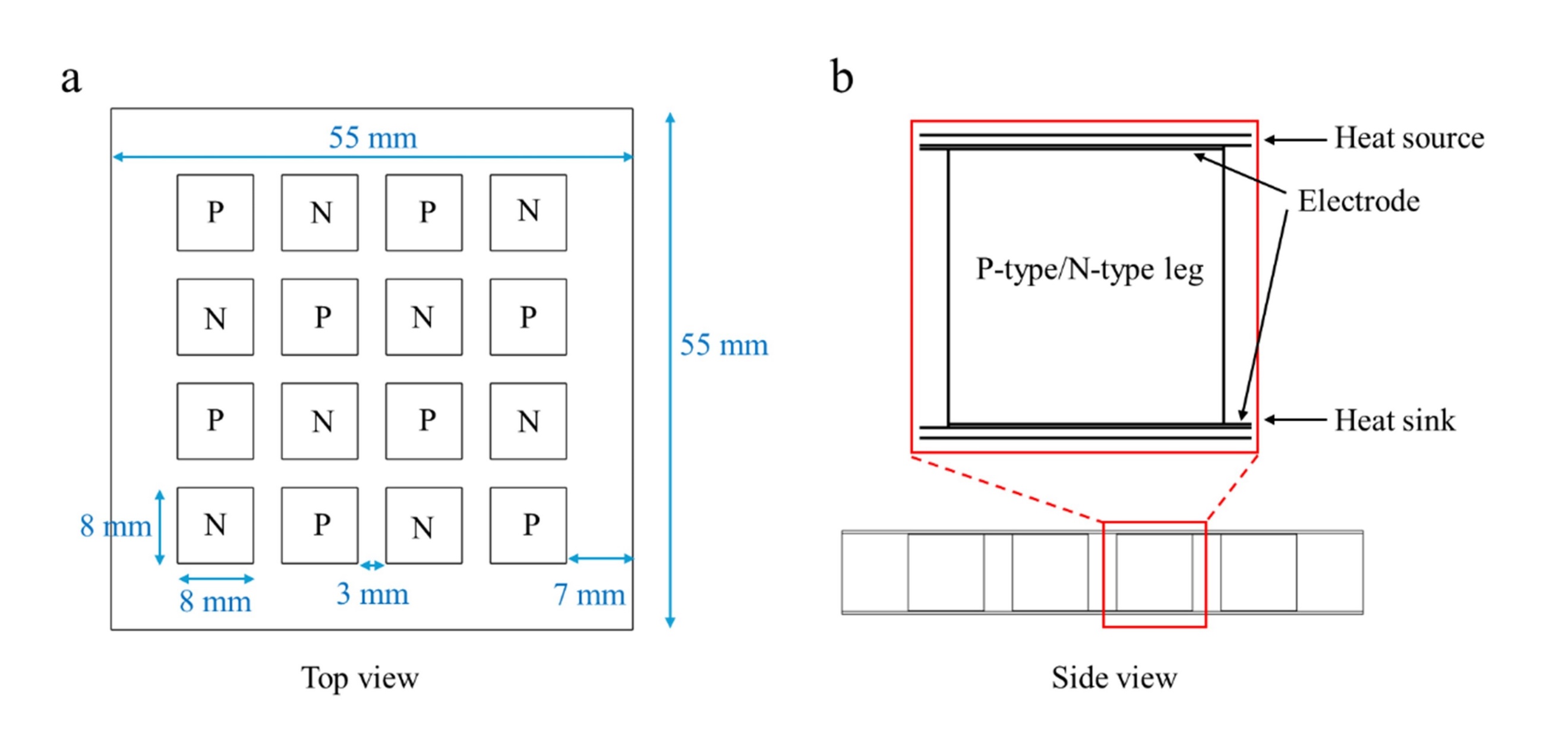


**Figure S19.** (a) Top view and (b) side view of the simulation model.

**
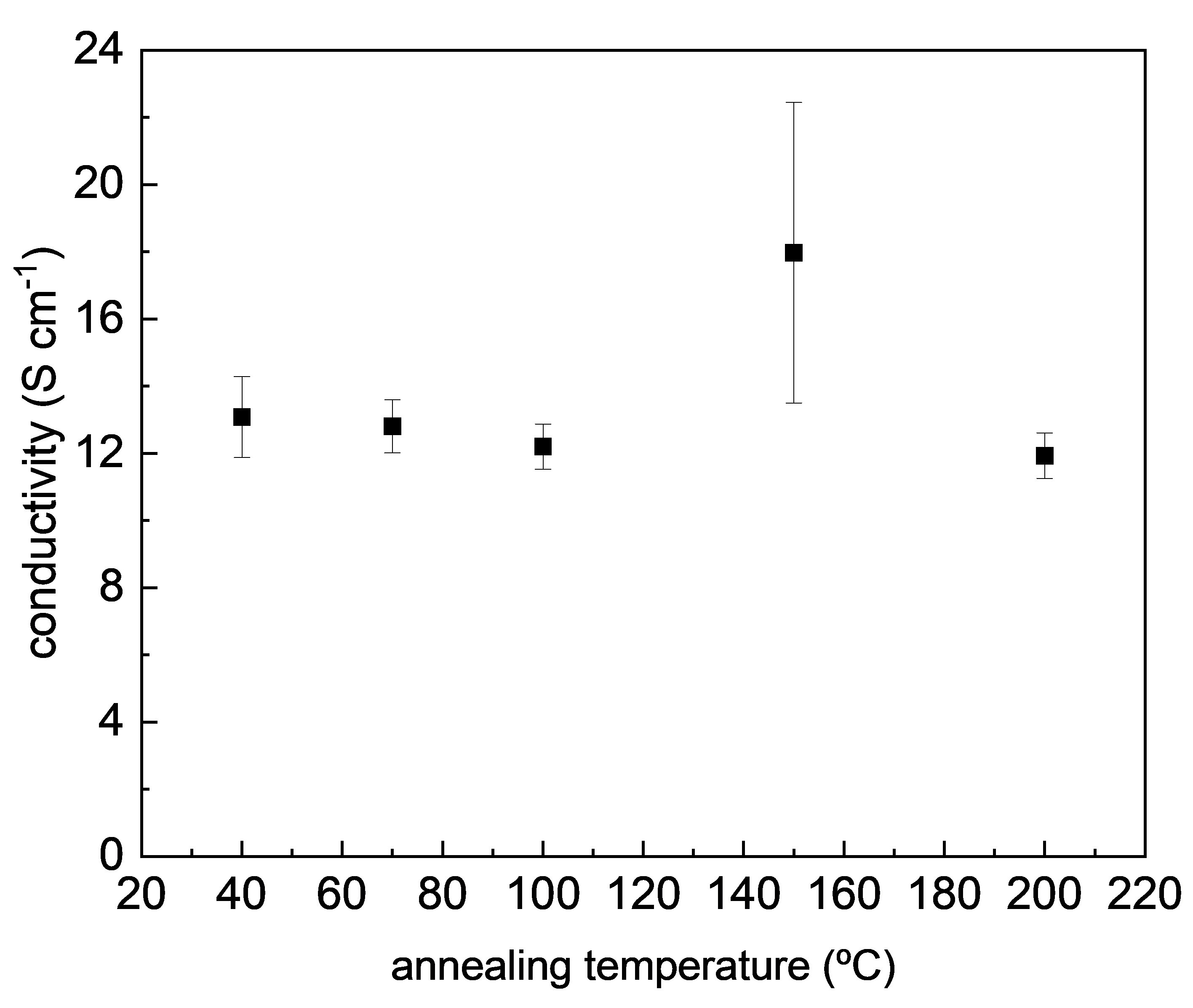
**

**Figure S20.** Bulk electrical conductivity of PBFDO coated yarns at different temperatures.
